# Association of non-alcoholic fatty liver disease with major adverse cardiovascular events: A systematic review and meta-analysis

**DOI:** 10.1038/srep33386

**Published:** 2016-09-16

**Authors:** Shunquan Wu, Fuquan Wu, Yingying Ding, Jun Hou, Jingfeng Bi, Zheng Zhang

**Affiliations:** 1 Research Center for Clinical and Translational Medicine, Beijing 302 Hospital, Beijing, China; 2 Department of General Surgery, 309th Hospital of PLA, Beijing, China; 3 Department of Medical Microbiology and Parasitology, Second Military Medical University, Shanghai, China

## Abstract

Increasing evidence connects non-alcoholic fatty liver disease (NAFLD) to cardiovascular disease (CVD). The aim of this study is to assess whether and to what extent the excess risk of CVD is conferred by NAFLD in a meta-analysis. We systematically searched PubMed, EmBase, Web of Science, and Cochrane Library for reports published between 1965 and July 3, 2015. Studies that reported data on association between NAFLD and adverse cardiovascular events or mortality were included. Thirty-four studies (164,494 participants, 21 cross-sectional studies, and 13 cohort studies) were included. NAFLD was not associated with overall mortality (HR = 1.14, 95% CI: 0.99–1.32) and CVD mortality (HR = 1.10, 95% CI: 0.86–1.41). However, NAFLD was associated with an increased risk of prevalent (OR = 1.81, 95% CI: 1.23–2.66) and incident (HR = 1.37, 95% CI: 1.10–1.72) CVD. For some specific CVDs, NAFLD was associated with an increased risk of prevalent (OR = 1.87, 95% CI: 1.47–2.37) and incident (HR = 2.31, 95% CI: 1.46–3.65) coronary artery disease (CAD), prevalent (OR = 1.24, 95% CI: 1.14–1.36) and incident (HR = 1.16, 95% CI: 1.06–1.27) hypertension, and prevalent (OR = 1.32, 95% CI: 1.07–1.62) atherosclerosis. In conclusion, the presence of NAFLD is associated with an increased risk of major adverse cardiovascular events, although it is not related to mortality from all causes or CVD.

Non-alcoholic fatty liver disease (NAFLD) is the leading cause of chronic liver disease worldwide, with a prevalence as high as 30% in the general population^1^. Obesity, hyperlipidemia, diabetes mellitus, the metabolic syndrome and insulin resistance have been established as risk factors for primary NAFLD^2^,^3^. The disease can progress to more aggressive forms of nonalcoholic steatohepatitis (NASH), which can progress to cirrhosis, end-stage liver disease, and eventually hepatocellular carcinoma^4^. Especially, as NAFLD was known to be related to metabolic syndrome, there was increasing attention for the clinical association between NAFLD and cardiovascular disease (CVD) morbidity and mortality, both in non-diabetic and type 2 diabetic individuals^5^,^6^,^7^. However, the connections between NAFLD and CVD were not consistent^8^, and the independent association of fatty liver and cardiovascular risk may persist or disappear after controlling for cardiovascular risk factors such as obesity, hypertension, or diabetes, suggesting the unclear role of fatty liver in CVD.

Although CVD mortality rates have decreased by as much as 50% in several developed countries since the 1970s, it still remains the most frequent cause of death worldwide^9^. As the high morbidity, mortality, and health care costs associated with CVD, it is crucial to investigate the association between NAFLD and adverse cardiovascular events for the purpose of prevention. In the present study, we attempted a large-scale synthesis of the available epidemiological evidence under a systematic review and meta-analysis to determine the association between NAFLD and major patient-important cardiovascular outcomes.

## Results

### Characteristics of included studies and participants

Our initial search yielded 1641 records, of which 1560 remained after removal of duplicates (Figure S1). Thirty-four studies (164,494 participants) met our inclusion criteria and were included in our analysis (Table 1). All relevant studies identified were published in or translated into the English language. Twenty-one studies (44,279 participants) were cross-sectional and evaluated the association of NAFLD with prevalent adverse cardiovascular events^9^,^10^,^11^,^12^,^13^,^14^,^15^,^16^,^17^,^18^,^19^,^20^,^21^,^22^,^23^,^24^,^25^,^26^,^27^,^28^,^29^; 13 studies (120,215 participants) were prospective cohorts and evaluated the association of NAFLD or NASH with new-onset adverse cardiovascular events or mortality^30^,^31^,^32^,^33^,^34^,^35^,^36^,^37^,^38^,^39^,^40^,^41^,^42^. Twenty-five studies (109,639 participants) used hospital-based design^9^,^10^,^11^,^12^,^13^,^14^,^15^,^16^,^17^,^19^,^20^,^22^,^23^,^24^,^25^,^26^,^29^,^32^,^33^,^36^,^37^,^38^,^40^,^41^,^42^ and nine studies (54,855 participants) used population-based design^18^,^21^,^27^,^28^,^30^,^31^,^34^,^35^,^39^. NAFLD or NASH was defined by ultrasound in 25 studies (129,755 participants)^9^,^10^,^11^,^12^,^13^,^14^,^15^,^16^,^17^,^18^,^21^,^23^,^24^,^25^,^26^,^28^,^29^,^30^,^33^,^34^,^36^,^37^,^39^,^40^,^42^, by CT images in five studies (14,738 participants)^19^,^20^,^22^,^27^,^41^, by liver biopsy in three studies (8,716 participants)^31^,^32^,^38^, and by liver enzyme elevation in one study (11,285 participants)^35^. Data were available for analysis for risks of overall mortality, CVD mortality, and prevalent/incident overall CVD according to participants with NAFLD compared with those without NAFLD. We also assessed the risks of some specific CVDs, such as prevalent/incident coronary artery disease (CAD), prevalent/incident hypertension, and prevalent atherosclerosis. The risks of overall mortality, CVD mortality, and incident CVD according to participants with NASH compared with those without NASH were also assessed. Overall, the quality of the included studies was good: the median (range) NOS score was 4 (3–5) stars for cross-sectional studies and 8 (5–9) stars for cohort studies. The quality assessments of the included studies were presented in Tables S2 and S3 in detail.

Table S4 shows the WMDs in baseline risk factor levels among the included population, compared NAFLD participants with non-NAFLD participants, from those studies for which individual participant data were available. Compared with individuals without NAFLD, those with NAFLD had significantly higher BMI (WMD = 2.82, 95% CI: 2.43–3.21), waist circumference (WMD = 8.62, 95% CI: 7.70–9.54), systolic blood pressure (WMD = 6.09, 95% CI: 4.82–7.35), diastolic blood pressure (WMD = 3.77, 95% CI: 2.83–4.71), total cholesterol (WMD = 11.57, 95% CI: 8.54–14.61), low-density lipoprotein cholesterol (WMD = 7.62, 95% CI: 4.13–11.11), triglycerides (WMD = 52.27, 95% CI: 45.62–58.91), fasting glucose (WMD = 8.34, 95% CI: 7.00–9.69), alanine aminotransferase (WMD = 14.03, 95% CI: 10.98–17.08), aspartate aminotransferase (WMD = 6.04, 95% CI: 4.48–7.60), γ-glutamyltranspeptidase (WMD = 13.32, 95% CI: 9.88–16.76), and mean carotid intimal-medial thickness (WMD = 0.06, 95% CI: 0.02–0.11), and significantly lower high-density lipoprotein cholesterol (WMD = −5.62, 95% CI: −6.63 to −4.62).

### NAFLD and risk of mortality

#### Overall mortality

**Figure 1:**
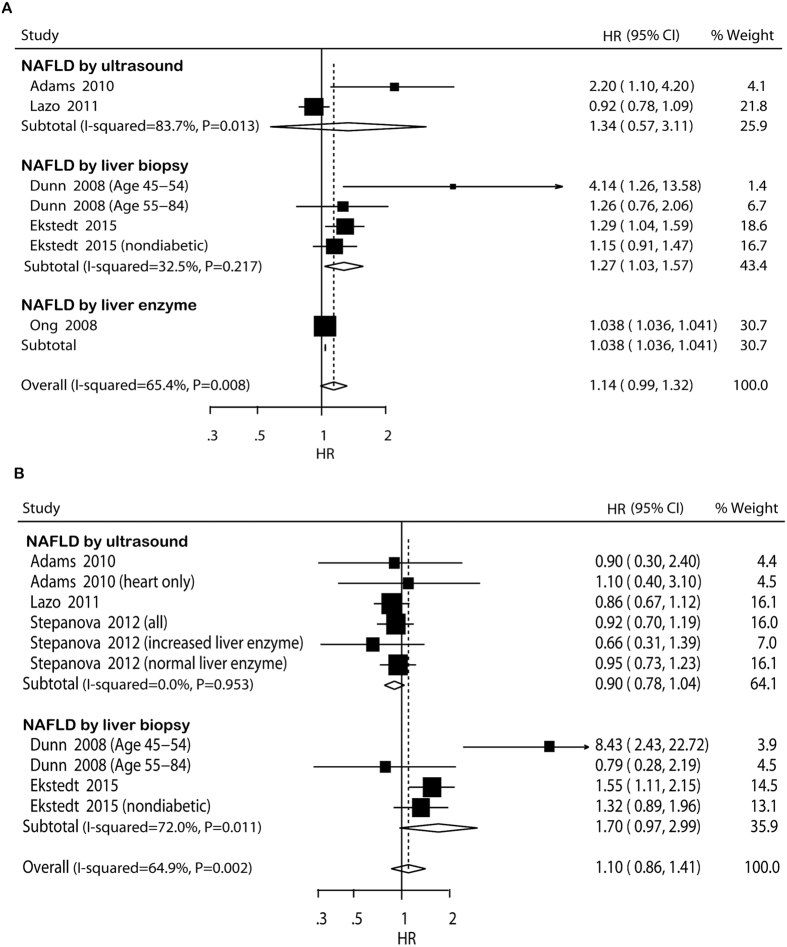
Forest plot of comparison. NAFLD versus non-NAFLD, outcome: overall mortality (**A**) and cardiovascular disease mortality (**B**) based on cohort studies. Studies assessing NAFLD by ultrasound, liver biopsy or liver enzyme were considered separately.

Data on overall mortality were available for analysis in seven comparisons from five cohort studies. The pooled analysis showed that there was no statistically significant difference in overall mortality between NAFLD participants and non-NAFLD participants (HR = 1.14, 95% CI: 0.99–1.32) (Fig. 1A). The *I*^*2*^ statistic for heterogeneity between studies was 65.4%, with *P* value for the χ^2^ test 0.008, suggesting substantial between-study heterogeneity. In the subgroup analyses that had more than one comparison, individuals with NAFLD had significantly higher risk of overall mortality than those without NAFLD in studies using hospital-based study design and with relatively low study quality (Table S5). Sensitivity analysis indicated that the non-significant difference in overall mortality was not materially changed in the leave-one-out analyses by omitting one study in turn except for the study of Lazo and colleagues^34^, with pooled HRs range from 1.10 (95% CI: 0.96–1.25) to 1.25 (95% CI: 0.99–1.59) (Figure S2A).

#### CVD mortality

Data on CVD mortality were available for analysis in ten comparisons from five cohort studies. Similarly, meta-analysis did not show significant difference in CVD mortality between NAFLD participants and non-NAFLD participants (HR = 1.10, 95% CI: 0.86–1.41) (Fig. 1B). Potential heterogeneity was explored among the individual studies (*I *^2^ = 64.9%, *P* = 0.002). In the subgroup analyses, the pooled HRs did not differ significantly by most of the study-level factors except for studies with hospital-based design (Table S5). Univariate meta-regression analysis showed that the regression coefficients of publication year (*P* = 0.032), study design (*P* = 0.007), follow-up duration (*P* = 0.079), BMI (*P* = 0.001), and presence of diabetes (*P* = 0.054) were significant at the level of 0.1, and these five covariates were entered into the multivariate meta-regression analysis. After including these five covariates in the model, the τ^2^ changed from 0.0539 to 0.01191, which means that 77.90% of heterogeneity between the studies can be explained by these covariates. Sensitivity analysis indicated that the non-significant difference in CVD mortality was not materially changed in the leave-one-out analyses by omitting one study in turn, with pooled HRs range from 1.02 (95% CI: 0.86–1.21) to 1.16 (95% CI: 0.87–1.55) (Figure S2B).

### NAFLD and prevalent/incident CVD

#### Cross-sectional studies

**Figure 2:**
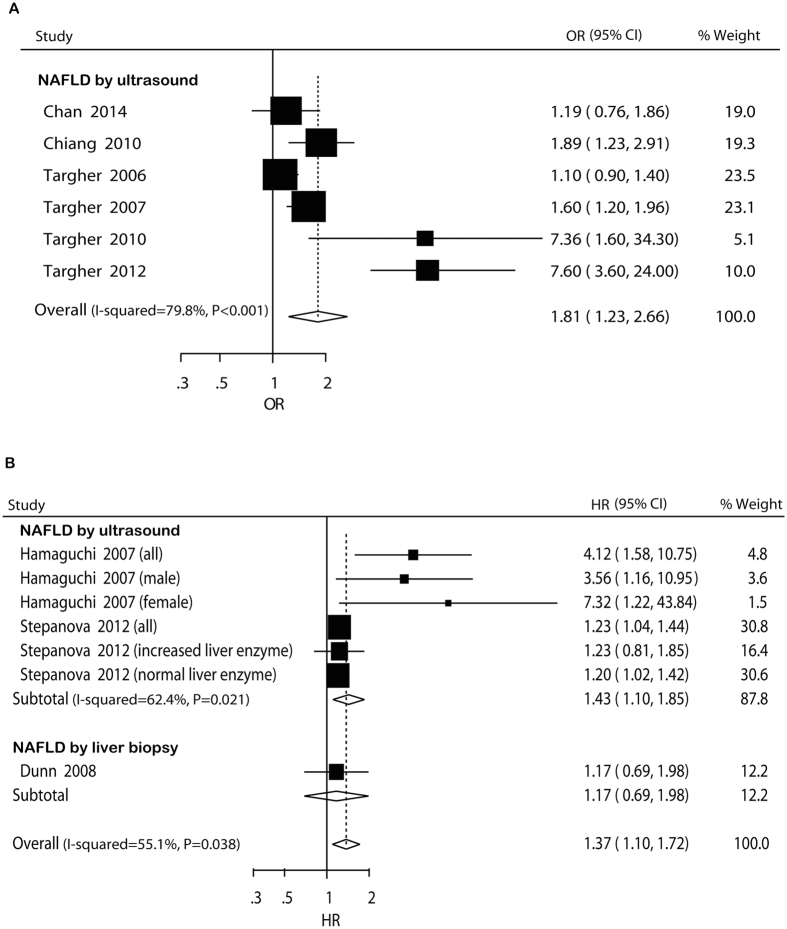
Forest plot of comparison. NAFLD versus non-NAFLD, outcome: prevalent cardiovascular disease in cross-sectional studies (**A**) and incident cardiovascular disease in cohort studies (**B**). Studies assessing NAFLD by ultrasound or liver biopsywere considered separately.

In cross-sectional studies, pooled OR for the presence of CVD of NAFLD versus non-NAFLD was 1.81 (95% CI: 1.23–2.66) (Fig. 2A). There was potential heterogeneity among the individual studies (*I*^ 2^ = 79.8%, *P* < 0.001). Subgroup analyses indicated that the significantly higher risk was not seen participants with mean age over 50 years, in studies including exclusively Asian participants, with relatively low study quality, and not adjusting for age or BMI/obesity or smoking (Table S5). Sensitivity analysis indicated that the significantly higher risk was not materially changed in the leave-one-out analyses by omitting one study in turn, with pooled ORs range from 1.48 (95% CI: 1.09–1.99) to 2.15 (95% CI: 1.34–3.46) (Figure S2C).

#### Cohort studies

In cohort studies, pooled HR for incident CVD of NAFLD versus non-NAFLD was 1.37 (95% CI: 1.10–1.72) (Fig. 2B). Potential heterogeneity among the studies was observed (*I*^ 2^ = 55.1%, *P* = 0.038). The pooled HRs did not differ significantly in the subgroup analyses that had more than one comparison (Table S5). The leave-one-out analyses indicated that the significantly higher risk was not materially changed by omitting one study in turn, with pooled HRs range from 1.26 (95% CI: 1.07–1.49) to 1.62 (95% CI: 1.11–2.35) (Figure S2D).

### NAFLD and prevalent/incident CAD

#### Cross-sectional studies

**Figure 3:**
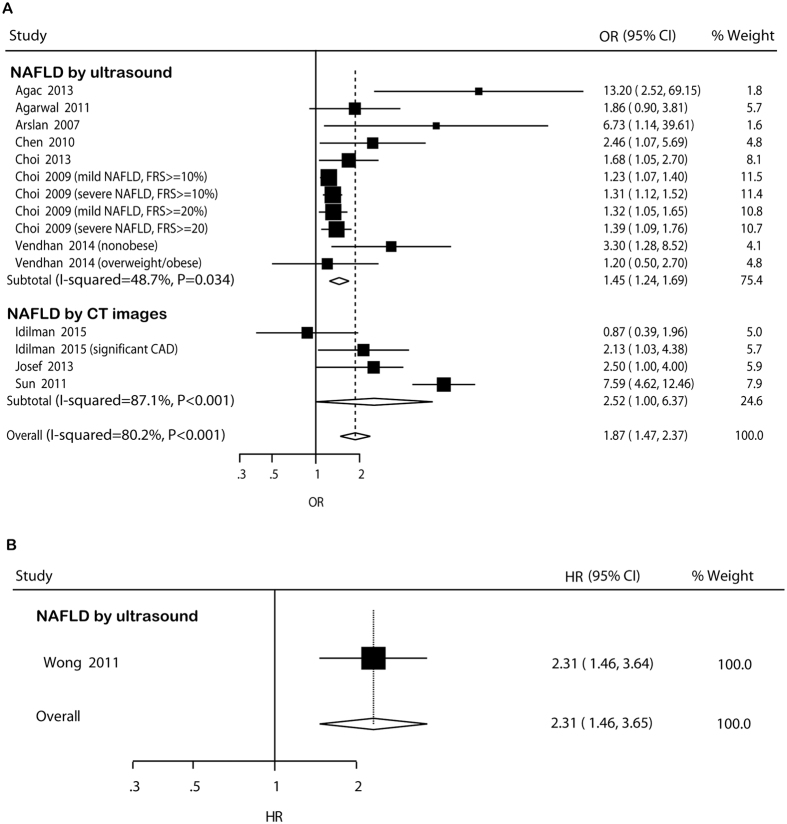
Forest plot of comparison. NAFLD versus non-NAFLD, outcome: prevalent coronary artery disease in cross-sectional studies (**A**) and incident coronary artery disease in cohort studies (**B**). Studies assessing NAFLD by ultrasound or CT images were considered separately.

In cross-sectional studies, pooled OR for prevalent CAD of NAFLD versus non-NAFLD was 1.87 (95% CI: 1.47–2.37) (Fig. 3A). There was potential heterogeneity among the individual studies (*I*^ 2^ = 80.2%, *P* < 0.001). In the subgroup analyses, the higher risk was not significant in studies using population-based design and including exclusively diabetic participants (Table S5). In the univariate meta-regression analysis, the regression coefficients of age (*P *= 0.035), male percent (*P *= 0.061), study design (*P *= 0.092), ethnicity (*P *= 0.078), and study quality (*P *= 0.013) were significant at the level of 0.1, and these five covariates were entered into the multivariate meta-regression analysis. After including these five covariates in the model, the τ^2^ changed from 0.06891 to 0.01922, which means that 72.10% of heterogeneity between the studies can be explained by these covariates. Sensitivity analysis indicated that the significantly higher risk was not materially changed in the leave-one-out analyses by omitting one study in turn, with pooled ORs range from 1.49 (95% CI: 1.28–1.75) to 2.02 (95% CI: 1.52–2.69) (Figure S2E).

#### Cohort studies

Only one cohort study was available for assessment of association between NAFLD and incident CAD. The HR reported by the study was 2.31 (95% CI: 1.46–3.65) (Fig. 3B).

### NAFLD and prevalent/incident hypertension

#### Cross-sectional studies

**Figure 4:**
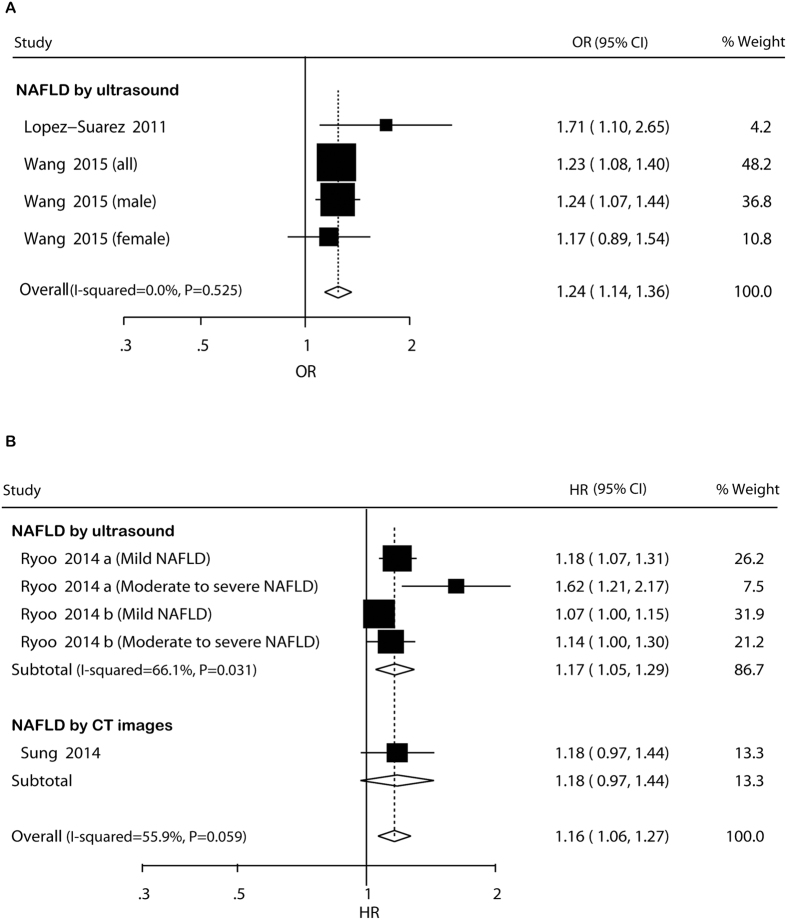
Forest plot of comparison. NAFLD versus non-NAFLD, outcome: prevalent hypertension in cross-sectional studies (**A**) and incident hypertension in cohort studies (**B**). Studies assessing NAFLD by ultrasound or CT images were considered separately.

In cross-sectional studies, pooled OR for prevalent hypertension of NAFLD versus non-NAFLD was 1.24 (95% CI: 1.14–1.36) (Fig. 4A). There was no potential heterogeneity among the individual studies (*I*^ 2^* *= 0.0%, *P *= 0.525). The magnitude and direction of the associations were unaltered across studies in the subgroup analyses (Table S5). The leave-one-out analyses indicated that the significantly higher risk was not materially changed by omitting one study in turn, with pooled ORs range from 1.23 (95% CI: 1.12–1.35) to 1.26 (95% CI: 1.10–1.45) (Figure S2F).

#### Cohort studies

In cohort studies, pooled HR for incident hypertension of NAFLD versus non-NAFLD was 1.16 (95% CI: 1.06–1.27) (Fig. 4B). Potential heterogeneity among the studies was observed (*I*^ 2^* *= 55.9%, *P *= 0.059). In the subgroup analyses, the higher risk was not significant in studies not adjusting for age or BMI/obesity or smoking (Table S5). Sensitivity analysis indicated that the significantly higher risk was not materially changed in the leave-one-out analyses by omitting one study in turn, with pooled HRs range from 1.11 (95% CI: 1.06–1.17) to 1.20 (95% CI: 1.09–1.33) (Figure S2G).

### NAFLD and prevalent atherosclerosis

**Figure 5:**
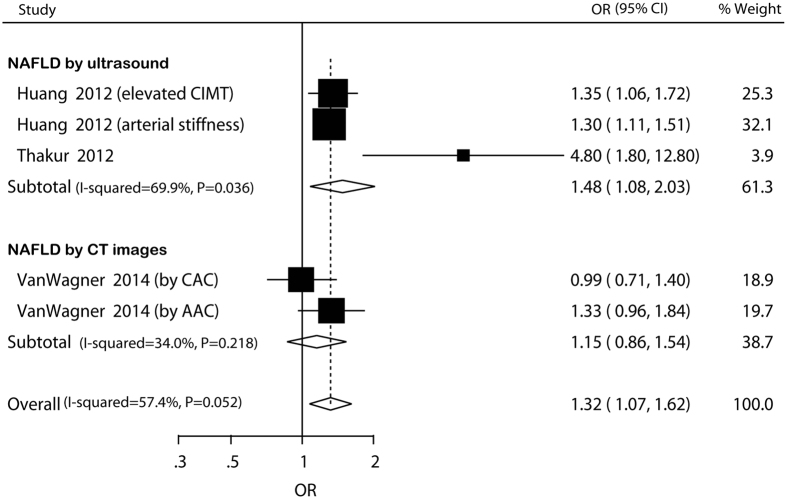
Forest plot of comparison. NAFLD versus non-NAFLD, outcome: prevalent atherosclerosis based on cross-sectional studies. Studies assessing NAFLD by ultrasound or CT images were considered separately.

Data for association between NAFLD and atherosclerosis were only available in cross-sectional studies. Pooled OR for prevalent atherosclerosis of NAFLD versus non-NAFLD was 1.32 (95% CI: 1.07–1.62) (Fig. 5). There was no potential heterogeneity among the individual studies (*I*^ 2^* *= 34.0%, *P *= 0.218). In the subgroup analyses, the higher risk was not significant in studies including exclusively non-Asian participants and not adjusting for age or BMI/obesity or smoking (Table S5). Sensitivity analysis indicated that the higher risk was not materially changed in the leave-one-out analyses by omitting one study in turn except for one comparison in the study of Huang and colleagues^18^, with pooled ORs range from 1.27 (95% CI: 1.14–1.43) to 1.40 (95% CI: 1.13–1.75) (Figure S2H).

### NASH, mortality and CVD incidence

**Figure 6:**
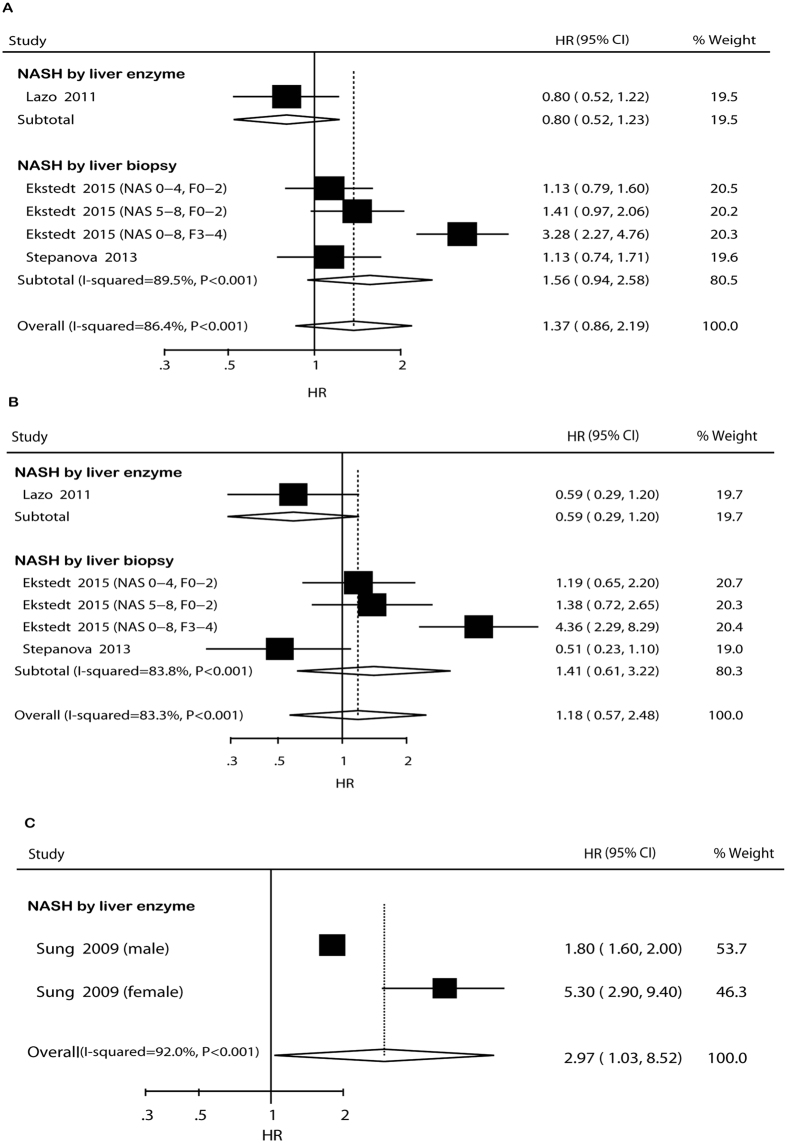
Forest plot of comparison. NASH versus non-NASH, outcome: overall mortality (**A**), cardiovascular disease mortality (**B**) and incident cardiovascular disease (**C**) based on cohort studies. Studies assessing NASH by ultrasound or liver biopsy were considered separately.

Data for association between NASH and adverse cardiovascular events were only available for assessment of overall mortality, CVD mortality, and CVD incidence in cohort studies. Meta-analyses indicated that NASH was not associated with overall mortality (HR* *= 1.37, 95% CI: 0.86–2.19, *I*^ 2^* *= 86.4%, *P* < 0.001) and CVD mortality (HR* *= 1.18, 95% CI: 0.57–2.48, *I*^ 2^* *= 83.3%, *P* < 0.001) but significantly increased the incident CVD risk (HR* *= 2.97, 95% CI: 1.03–8.52, *I* ^2^ **= 92.0%, *P* < 0.001) (Fig. 6). Subgroup analyses and sensitivity analyses were only conducted for overall mortality and CVD mortality, because there were only two comparisons for CVD incidence. In the subgroup analyses, the pooled HRs did not differ significantly (Table S5). Sensitivity analysis indicated that the non-significant risks were not materially changed in the leave-one-out analyses by omitting one study in turn (Figure S2I,J).

### Publication bias

There was no potential publication bias in most of our analyses as assessed by funnel plots, Egger’s regression test and Begg-Mazumdar test (Figure S3). Egger’s regression test (*P *= 0.020) indicated there was potential publication bias when assessing NAFLD and CVD incidence in cohort studies (Figure S3D). After using the trim and fill approach, one study was filled and the pooled result did not reverse (HR* *= 1.36, 95% CI: 1.06–1.74) (Figure S3E). Both Egger’s regression test (*P *= 0.013) and Begg-Mazumdar test (*P *= 0.012) indicated there was potential publication bias when assessing NAFLD and CAD prevalence in cross-sectional studies (Figure S3F). After using the trim and fill approach, six studies were filled and the pooled result did not reverse (OR* *= 1.36, 95% CI: 1.04–1.77) (Figure S3G).

## Discussion

The main results of our meta-analysis are the following: (1) NAFLD was not associated with overall mortality and CVD mortality; (2) NAFLD was associated with an increased prevalence and/or incidence of other adverse cardiovascular events, including CVD, CAD, hypertension, and atherosclerosis; (3) NASH was not associated with overall mortality and CVD mortality but was associated with an increased incidence of CVD. These results are important given the high prevalence of NAFLD in the general population and the concerns raised by the adverse metabolic profile associated with this disease and NASH.

Based on the current evidence from the literature, the association between NAFLD and adverse cardiovascular events is mixed. Several researchers reported that NAFLD is associated with CVD in diabetic patients^24^,^33^,^42^,^43^, but others did not confirm this finding^8^,^44^. We believe the explanation for this contrasting finding lies in important differences in the study populations, sample size, study design, study duration and disease ascertainment methods. The value of the current meta-analysis compensates for the individual lack of precision in most of the studies, a problem that was alleviated by pooling the data of all the studies.

In our study, we found no association between NAFLD or NASH and deaths from all causes or CVD. This result was similar with a cohort study from Sweden. This study followed up a cohort of 144 patients with NAFLD for over 13.7 years, and found patients with NAFLD had similar survival to the general Swedish population (matched for age and gender). However, their study found the risk of death was increased in patients with NASH^45^. Similarly, two other studies also found no evidence of an increased risk of death among patients with NAFLD compared with the general population of either the United Kingdom or Denmark^46^,^47^. However, there was no adjustment for potential confounders in these studies. In contrast, one study reported an increased risk of death from all causes among 1804 patients with fatty liver, with standardized mortality ratio compared with general Danish population of 2.6; but this study was not able to adjust for confounders either^48^. The potential confounders in these studies make it difficult to draw firm conclusions about the impact of NAFLD and NASH on mortality.

In addition, our study indicated that NAFLD was an independent risk factor in determining cardiovascular events (including CVD, CAD, hypertension, and atherosclerosis) by pooling the multiple-adjusted data together, and NASH was an independent risk factor in determining CVD. Hamaguchi and colleagues^49^ showed NAFLD is strongly related to metabolic syndrome and therefore shares many risk factors with cardiovascular disease, suggesting a close relationship between NAFLD and adverse cardiovascular events. Some other mechanisms may explain the higher risks of cardiovascular events in patients with NAFLD. The disease is associated with a proatherogenic lipid profile^50^ and increased production of pro-inflammatory cytokines including IL-6^51^. Experimental researches also showed that the degree of liver injury and chronic inflammation play an important role in the pathogenesis of atherosclerosis^52^. In addition, it has been reported that patients with NAFLD had larger carotid intima-media thickness^53^,^54^, increased prevalence of endothelial dysfunction^55^ and calcified and noncalcified coronary plaques^56^, providing evidence of cardiovascular damage in NAFLD. Other potential mechanisms by which NAFLD increases cardiovascular risk are increased oxidative stress, prothrombotic state and systemic inflammation^57^.

Our study showed that NASH, a more severe stage of NAFLD, was associated with higher risks in overall mortality, CVD mortality, and incident CVD with larger HRs, compared with NAFLD, although the risks were not significant in overall mortality and CVD mortality. A previous study showed that NASH is associated with a more severe inflammatory and insulin-resistant state that promotes atherosclerosis^58^. Singh *et al.*^59^ demonstrated that the annual fibrosis progression rate in patients with NASH (0.14) was higher than that in patients with non-alcoholic fatty liver (0.07). On the other hand, long-standing NASH can result advanced fibrosis and may indirectly reflect exposure to risk factors for cardiovascular disease. Histology-based studies have also suggested that cardiovascular disease is mainly associated with more severe forms of NAFLD^45^,^60^. Subgroup analysis by mean age of the participants showed that in the analyses which had more than one comparison, elderly participants (mean age ≥50 years) had higher cardiovascular risks with larger point estimates, suggesting further increased risk involving an age-related mechanisms. Frith and colleagues^61^ indicated that elderly patients with NAFLD had greater fibrosis in biopsy and significantly more cardiovascular risk factors, including obesity, diabetes, hypertension, and hyperlipidemia. Another study also reported that fat may become dysfunctional and redistribute from subcutaneous to intra-abdominal visceral depots in old-age patients^62^, and the fat redistribution in the elderly has been shown to be related to increased traditional cardiovascular risk factors such as hypertension, diabetes, central obesity, atherosclerosis and dyslipidemia.

Recently, Targher *et al.*^63^ also conducted a meta-analysis on NAFLD and risk of incident CVD. The differences between their study and ours are that they pooled prospective and retrospective studies together to calculate the risk of incident CVD, while our study included cross-sectional and prospective cohort study and pooled them separately to calculate the risk of prevalent and incident CVD. In addition, their study only reported the risk of overall CVD, while our study not only reported the risk of overall CVD but also reported the risks of some specific CVDs.

This meta-analysis provides the most definitive and convincing evidence so far of NAFLD-related risk of cardiovascular events. The findings were robust and applicable across a broad range of populations. However, the study has several limitations. First, the most pooled analyses revealed heterogeneity among studies. Although subgroup analysis and meta-regression analysis gave some clues to explain the heterogeneity, these are unlikely to have fully accounted for heterogeneity. Therefore, the results of the meta-analysis must be interpreted with caution. Because of potential additional heterogeneity in the populations, designs, and analyses of the various studies, we assumed that the true effect being estimated would vary between studies, in addition to the usual sampling variation in the estimates. To account for the heterogeneity, we used the random-effect model to combine the results of the primary studies. The random-effect approach provides some allowance for heterogeneity in studies beyond sampling error. This does not necessarily rule out the effect of heterogeneity between the studies, but one can expect a very limited influence because of it. Second, the meta-analysis is based on observational studies, which leaves the possibility that residual confounding factors, including measurement errors, affect the relation between NAFLD and adverse cardiovascular events. Third, data were only available for analysis for risks of overall mortality, CVD mortality, prevalent/incident CVD, prevalent/incident CAD, prevalent/incident hypertension, and prevalent atherosclerosis, and other adverse cardiovascular events are not analyzed because studies on other adverse cardiovascular events are quite few for solid meta-analysis. More studies are needed to confirm the relationship between NAFLD and risk of other specific cardiovascular diseases. Fourth, there were limited studies in several subgroup analyses, which may lead to low statistical power in these analyses. Another important limitation of these data, which warrants further investigation by future studies, was that a standard definition of diabetes was not used across studies and ascertainment of diabetes probably varied between studies. Additionally, although we did subgroup analysis by the presence of diabetes, data on exclusively diabetic participants and non-diabetic participants were quite few. Further studies are needed to examine the potential contribution that the presence of diabetes might have on the excess risk of adverse cardiovascular events.

In conclusion, our analysis shows that the presence of NAFLD is not associated with overall mortality and CVD mortality but is associated with an increased risk of other adverse cardiovascular events, including CVD, CAD, hypertension, and atherosclerosis. Future studies should evaluate strategies and interventions to prevent cardiovascular disease progression in individuals with NAFLD.

## Methods

### Search strategy and eligibility criteria

We followed the PRISMA guidelines^64^ to complete the meta-analysis. Two investigators (SW and YD) conducted a systematic literature search of PubMed, EmBase, Web of Science, and Cochrane Library for identification of articles published between 1965 and July 3, 2015, using a combined text and MeSH heading search strategy with the terms: “non-alcoholic fatty liver disease”, “nonalcoholic fatty liver disease”, “NAFLD”, “non-alcoholic steatohepatitis”, “NASH”, “fatty liver”, “liver fat”, “steatosis”, “cardiovascular diseases”, “atherosclerosis”, “stroke”, “atrial fibrillation”, “overall mortality”, “coronary artery disease”, “coronary heart disease”, “hypertension”, and “mortality”. The search was restricted to studies in human beings and no language restriction was imposed. We also checked the reference lists of identified reports for other potentially relevant studies. We contacted the authors of the included studies to ask them for additional information and unpublished data.

We included studies that met the following criteria: participants aged 18 years or older; cross-sectional design, prospective design, or retrospective design; the association between NAFLD and adverse cardiovascular events or mortality was assessed; and reported data on odds ratios (ORs) or hazard ratios (HRs) with confidence intervals (CIs) or sufficient information to calculate these, for the association between NAFLD and adverse cardiovascular events or mortality. Studies were excluded if they did not provide information to calculate the point estimate, did not make comparison between NAFLD and adverse cardiovascular events or mortality, or were review studies. Articles that clearly did not meet inclusion criteria were rejected on initial review. If uncertainty existed, the full text of the article was reviewed. Two reviewers (FW and JH) independently assessed all potentially relevant studies for inclusion. Disagreements were resolved by consensus.

### Data extraction and study quality evaluation

Study characteristics were extracted independently by two researchers (PM and YH). We extracted risk estimates (95% CI) for different genders and disease severities separately when possible. If a study reported more than one measure of adverse cardiovascular events, each adverse cardiovascular event was extracted separately. The most adjusted estimate was included when a study reported more than one risk estimate. The quality of each study was assessed by two researchers (FW and PM), using the Newcastle-Ottawa Scale (NOS) recommended by Wells and colleagues^65^. The quality of each included studies ranges from 1 to 9 stars for cohort and case-control studies and 1 to 5 stars for cross-sectional studies.

### Statistical analysis

Associations with continuous outcome variables were expressed as weighted mean differences (WMDs) with 95% CIs. For evaluation of the relative risk of adverse cardiovascular events, the effect size was estimated as ORs or HRs with 95% CIs, according to NAFLD patients *vs* non-NAFLD patients reported in each study, using non-NAFLD patients as the reference group. The impact of NAFLD histological subtypes (NASH) on adverse cardiovascular events was also examined.

We used the random-effect model in this meta-analysis to take into account heterogeneity among studies, because the study design and measuring time were different across studies^66^. The *I*-squared (*I*^ 2^) statistic and *Q*-statistic were used to explore the heterogeneity among studies. Large *I*^ 2^ (>50%) or *P* < 0.1 for *Q*-statistic suggests substantial heterogeneity among studies. We separately analyzed cross-sectional and longitudinal studies. Moreover, the results of studies defining NAFLD by ultrasound, liver biopsy, CT images, or liver enzyme elevation are presented separately. We did several subgroup analyses: study design (population-based or hospital-based), mean age of the participants (≥50 years or <50 years), ethnicity (non-Asian or Asian), presence of diabetes (diabetic participants or non-diabetic participants or combined), study quality (high *vs* relatively low), and adjustment of some major risk factors (yes *vs* no). When eight or more comparisons were available, the effect of several variables including age, publication year, male percent, study design, follow-up duration (for cohort studies), body mass index (BMI), ethnicity, smoke, presence of diabetes, and study quality was assessed by meta-regression analysis to further evaluate the source and strength of heterogeneity. We also performed sensitivity analyses by removing each individual study from the meta-analysis^67^. Funnel plots were used to examine the presence of publication bias (ie, by plotting the natural log of the odds ratio against its standard error). We used Egger’s regression test and Begg-Mazumdar test to further assess publication bias. All statistical analyses were done with Stata Version 12.0 software (Stata Corp, College Station, TX).

## Additional Information

**How to cite this article**: Wu, S. *et al.* Association of non-alcoholic fatty liver disease with major adverse cardiovascular events: A systematic review and meta-analysis. *Sci. Rep.* **6**, 33386; doi: 10.1038/srep33386 (2016).

## Supplementary Material

Supplementary Information

## Figures and Tables

**Table 1:** Characteristics of the included studies.

Author	Country	Study characteristics	Duration of follow-up	CVD risk factors	Liver disease diagnosis and prevalence	Outcome and prevalence	Adjustments	Study quality^a^
Cross-sectional								
Agac^10^	Turkey	Hospital; n* *= 80; Mean age 61 y; Male 81%; Asian 100%	—	Smokers 24%; DM34%; HTN 45%;Mean BMI 28 kg/m^2^;Met Sy 59%	Ultrasound;NAFLD 81%	CAD incidence; 54%	Sex, age, BMI, waist circumference, smoking status, family history of CAD, TC, TG, HDL-C, LDL-C, ALT, serum creatinine, presence of HTN, DM, and Met Sy	4
Agarwal^11^	India	Hospital; n* *= 124; Mean age 59 y; Male 60%; Asian 100%	—	Smokers 14%; DM100%; HTN 68%;Mean BMI 27 kg/m^2^;Met Sy 41%	Ultrasound;NAFLD 57%	CAD incidence; 54%	Age	3
Arslan^12^	Turkey	Hospital; n* *= 92; Mean age 57 y; Male 71%; Asian 100%	—	Smokers 50%; DM0%; HTN 58%;Mean BMI 28 kg/m^2^;Met Sy 45%	Ultrasound;NAFLD 71%	CAD incidence; 47%	Age, male sex, LDL-C, BMI, smoking history, and individual components of the Met Sy	4
Chan^13^	Malaysia	Hospital; n* *= 399; Mean age 63 y; Male 43%; Asian 100%	—	Smokers 4%; DM100%; HTN 91%;Mean BMI 28 kg/m^2^; Met Sy 95%	Ultrasound;NAFLD 50%	CVD incidence; 27%	None	3
Chen^14^	Taiwan, China	Hospital; n* *= 295; Mean age 53 y; Male 66%; Asian 100%	—	Smokers 21%; DM10%; HTN 30%;Mean BMI 25 kg/m^2^;Met Sy NA	Ultrasound;NAFLD 41%	CAD incidence; 13%	Sex, age, BMI, smoking, HTN, DM, FGF, TC, TG, HDL, LDL, ALT, AST, SUA, and gallbladder stones	5
Chiang^15^	Taiwan, China	Hospital; n* *= 724; Mean age 49 y; Male 93%; Asian 100%	—	Smokers 23%; DM6%; HTN 18%;Mean BMI 24 kg/m^2^;Met Sy 15%	Ultrasound;NAFLD 52%	CVD incidence; 27%	Age, elevated hsCRP level, Met Sy, HTN, DM, and dyslipidemia	5
Choi^16^	Korea	Hospital; n* *= 134; Mean age 63 y; Male 28%; Asian 100%	—	Smokers NA; DM16%; HTN 61%;Mean BMI 26 kg/m^2^;Met Sy NA	Ultrasound;NAFLD 60%	CAD incidence; 34%	Age, gender, glucose, HbA1c, BMI, TC, TG, and LDL	4
Choi^17^	Korea	Hospital; n* *= 17350; Mean age 49 y; Male 52%; Asian 100%	—	Smokers NA; DM6%; HTN 16%;Mean BMI kg/m^2^;Met Sy 21%	Ultrasound;NAFLD 33%	CAD incidence (FRS >=10%); 17% CAD incidence (FRS >=20%); 5%	Age, gender, BMI, WC, and Met Sy	5
Huang^18^	China	Population; n* *= 8632; Mean age 59 y; Male 31%; Asian 100%	—	Smokers 15%; DM18%; HTN 59%;Mean BMI 25 kg/m^2^;Met Sy 38%	Ultrasound;NAFLD 30%	Atherosclerosis incidence; NA	Age, sex, BMI, LDL-C, HOMA-IR score, regular exerciser, smoking status, drinking status, Met Sy, and prior histories of CVD	5
Idilman^19^	Turkey	Hospital; n* *= 273; Mean age 59 y; Male 47%; Asian 100%	—	Smokers 20%; DM100%; HTN 71%;Mean BMI 31 kg/m^2^;Met Sy 77%	CT images;NAFLD 22%	CAD incidence; 76% Significant CAD incidence; 35%	Age, gender, LDL-C levels, BMI, HTN and smoking status	4
Josef^20^	Israel	Hospital; n* *= 51; Mean age 52 y; Male 86%; Asian 100%	—	Smokers NA; DM0%; HTN 0%;Mean BMI 30 kg/m^2^;Met Sy 49%	CT images;NAFLD 57%	CAD incidence; 24%	Gender, age, smoking habits, Met Sy, DM, BMI, and levels of ALT, HDL and LDL-C, TG, and FG	4
Lopez-Suarez^21^	Spain	Population; n* *= 454; Mean age 61 y; Male 44%; Asian 0%	—	Smokers 10%; DM26%; HTN 46%;Mean BMI kg/m^2^;Met Sy NA	Ultrasound;NAFLD 39%	Hypertension incidence; 46%	Age, sex, sedentary lifestyle, smoking status, eGFR, DM, BMI, HDL-C, TG, and ALT	5
Sun^22^	China	Hospital; n* *= 542; Mean age 60 y; Male 65%; Asian 100%	—	Smokers 42%; DM27%; HTN 55%;Mean BMI 25 kg/m^2^;Met Sy 40%	CT images;NAFLD 46%	CAD incidence; 70%	Gender, age, previous myocardial infarction, TC, and AST	4
Targher^23^	Italy	Hospital; n* *= 800; Mean age 59 y; Male 54%; Asian 0%	—	Smokers 25%; DM100%; HTN NA;Mean BMI 27 kg/m^2^;Met Sy 80%	Ultrasound;NAFLD 50%	CVD incidence; 35%	Age, sex, DM duration, HbA, smoking history, LDL-C, GGT levels, use of medications, and MetS	5
Targher^24^	Italy	Hospital; n* *= 2392; Mean age 64 y; Male 56%; Asian 0%	—	Smokers 27%; DM100%; HTN NA;Mean BMI 28 kg/m^2^;Met Sy 83%	Ultrasound;NAFLD 70%	CVD incidence; 44%	Age, sex, BMI, smoking status, DM duration, A1C, LDL-C, and current use of medications	5
Targher^25^	Italy	Hospital; n* *= 202; Mean age 43 y; Male 51%; Asian 0%	—	Smokers 19%; DM100%; HTN NA;Mean BMI 25 kg/m^2^;Met Sy 39%	Ultrasound;NAFLD 55%	CVD incidence; 25%	Age, sex, DM duration, HbA, smoking status, LDL-C, Met Sy, BMI, SBP, HDL-C, TG, albuminuria, and medication use	4
Targher^9^	Italy	Hospital; n* *= 343; Mean age 45 y; Male 45%; Asian 0%	—	Smokers 22%; DM100%; HTN NA;Mean BMI 25 kg/m^2^;Met Sy 46%	Ultrasound;NAFLD 53%	CVD incidence; 31%	Age, gender, duration of DM, HbA, smoking status, alcohol consumption, physical activity level, family history of CVD, LDL-C, Met Sy, BMI, SBP, HDL-C, TG, current use of anti-hypertensive, lipid-lowering or anti-platelet medications, e-GFR, and albuminuria	4
Thakur^26^	India	Hospital; n* *= 80; Mean age 42 y; Male 68%; Asian 100%	—	Smokers NA; DMNA; HTN NA;Mean BMI 26 kg/m^2^;Met Sy 41%	Ultrasound;NAFLD 50%	Atherosclerosis incidence; 5%	Generalized and abdominal obesity, Met Sy, fasting insulin, dyslipidemias, systolic and diastolic blood pressure and hs-CRP	3
VanWagner^27^	The United States	Population; n* *= 2424; Mean age 50; Male 43%; Asian 0%	—	Smokers 14%; DM12%; HTN 33%;Mean BMI 31 kg/m^2^;Met Sy 28%	CT images;NAFLD 10%	Atherosclerosis incidence; 27%	Age, race, sex, study center, income level, educational level, alcohol intake, smoking status, physical activity score, DM status, SBP, TC, HDL, and treatments for HTN and dyslipidemia	5
Vendhan^28^	India	Population; n* *= 541; Mean age 43 y; Male 48%; Asian 100%	—	Smokers NA; DM0%; HTN NA;Mean BMI 24 kg/m^2^;Met Sy NA	Ultrasound;NAFLD 32%	CAD incidence; NA	Age, DM, hypercholesterolemia, HOMA-IR, and HTN	4
Wang^29^	Taiwan, China	Hospital; n* *= 8347; Mean age 38 y; Male 63%; Asian 100%	—	Smokers NA; DMNA; HTN 29%;Mean BMI 24 kg/m^2^;Met Sy NA	Ultrasound;NAFLD 48%	Hypertension incidence; 29%	Gender, age, BMI, hyperuricemia, AST, ALT, hypercholesterolemia, hypertriglyceridemia, and FG	4
Cohort								
Adams^30^	United States	Population; n* *= 337; Mean age 58 y; Male 49%; Asian 0%	10.5 years	Smokers 42%; DM100%; HTN 63%;Mean BMI 33 kg/m^2^;Met Sy NA	Ultrasound;NAFLD 34%	Overall mortality; 29% CVD mortality; 11% Heart disease mortality; 9%	Age, gender, obesity and date of DM diagnosis	9
Dunn^31^	The United States	Population; n* *= 8198; Mean age 52 y; Male 41%; Asian 0%	8.7 years	Smokers 20%; DM9%; HTN NA;Mean BMI NA;Met Sy 27%	Liver biopsy;NAFLD 14%	CVD incidence; 5% Overall mortality; 15% Overall mortality; 15%	Age, gender, race, SBP, DBP, WC, TC, HDL, TG, smoking, CRP, daily alcohol, physical activity, DM, and HMG-CoA reductase inhibitor use	8
Ekstedt^32^	Sweden	Hospital; n* *= 229; Mean age 49 y; Male 66%; Asian 0%	26.4 years	Smokers 21%; DM14%; HTN 57%;Mean BMI 28 kg/m^2^;Met Sy NA	Liver biopsy;NAFLD 65%	Overall mortality; 42% CVD mortality; 18%	NA	6
Hamaguchi^33^	Japan	Hospital; n* *= 1221; Mean age 48 y; Male 60%; Asian 100%	4.3 years	Smokers NA; DM0%; HTN NA;Mean BMI 23 kg/m^2^;Met Sy 13%	Ultrasound;NAFLD 19%	CVD incidence; 2%	Age, smoking, SBP, LDL-C, and Met Sy	6
Lazo^34^	The United States	Population; n* *= 11371; Mean age 43 y; Male 47%; Asian 0%	14.5 years	Smokers 27%; DM8%; HTN 23%;Mean BMI NA;Met Sy NA	Ultrasound;NAFLD 18%;NASH 4%	Overall mortality; 16% CVD mortality; 6%	Sex, race, education, smoking, alcohol consumption, physical activity, BMI, HTN, hypercholesterolaemia, and DM	9
Ong^35^	The United States	Population; n* *= 11285; Mean age NA; Male 47%; Asian 0%	8.7 years	Smokers NA; DM6%; HTN 23%;Mean BMI NA;Met Sy 25%	Liver enzyme elevation;NAFLD 7%	Overall mortality; 14%	Age, gender, race, education, income, BMI, HTN, and DM	9
Ryoo^36^	Korea	Hospital; n* *= 11350; Mean age 41 y; Male 100%; Asian 100%	2.8 years	Smokers 48%; DM3%; HTN 0%;Mean BMI 24 kg/m^2^;Met Sy 6%	Ultrasound;NAFLD 30%	Hypertension incidence; 58%	Age, HDL-C, log (hsCRP), serum creatinine, recent smoking status, regular exercise, MetS and DM	8
Ryoo^37^	Korea	Hospital; n* *= 22090; Mean age 42 y; Male 100%; Asian 100%	3.6 years	Smokers 42%; DM3%; HTN 0%;Mean BMI 24 kg/m^2^;Met Sy NA	Ultrasound;NAFLD 34%	Hypertension incidence; 17%	Age, BMI, TG, serum creatinine, AST, ALT, GGT, recent smoking status, regular exercise and DM	8
Stepanova^38^	The United States	Hospital; n* *= 289; Mean age 50 y; Male 39%; Asian 0%	12.5 years	Smokers NA; DM26%; HTN NA;Mean BMI 34 kg/m^2^;Met Sy NA	Liver biopsy;NASH 59%	Overall mortality; 40% CVD mortality; 11%	Age, gender, race, obesity, DM, and hyperlipidemia	6
Stepanova^39^	The United States	Population; n* *= 11613; Mean age 41 y; Male 48%; Asian 0%	14.3 years	Smokers 30%; DM5%; HTN NA;Mean BMI NA;Met Sy NA	Ultrasound;NAFLD 21%	CVD incidence; 31% CVD mortality; 4%	Age, sex, race, obesity, DM, smoking, and family history of CVD	7
Sung^40^	Korea	Hospital; n* *= 30172; Mean age 40 y; Male 60%; Asian 100%	NA	Smokers 17%; DM2%; HTN 5%;Mean BMI 23 kg/m^2^;Met Sy NA	Ultrasound;NASH 14%;Steatosis 9%	CVD incidence; 6%	Age, BMI, smoking and exercise habits	5
Sung^41^	Korea	Hospital; n* *= 11448; Mean age 41 y; Male 69%; Asian 100%	5 years	Smokers 49%; DM2%; HTN 0%;Mean BMI 24 kg/m^2^;Met Sy NA	CT images;NAFLD 38%	Hypertension incidence; 8%	Age, sex, alcohol consumption, smoking status, exercise, SBP, BMI, DM status, GGT, HOMA-IR, eGFR,and change in BMI	9
Wong^42^	Hongkong, China	Hospital; n* *= 612; Mean age 63 y; Male 71%; Asian 100%	1.6 years	Smokers 51%; DM31%; HTN 66%;Mean BMI 25 kg/m^2^;Met Sy NA	Ultrasound;NAFLD 58%	CAD incidence; 76%	Age, gender, smoking, alcohol, DM, HTN, SBP, DBP, BMI, WC, FG, TC, HDL-C, LDL-C, TG, creatinine, and ALT	7

^a^The quality of each included studies ranges from 1 to 9 stars for cohort studies and 1 to 5 stars for cross-sectional studies, based on the Newcastle-Ottawa Scale.

ALT, alanine aminotransferase; AST, aspartate aminotransferase; BMI, body mass index; CAD, coronary artery disease; CT, computed tomography; CVD, cardiovascular disease; DBP, diastolic blood pressure; DM, diabetes mellitus; eGFR, estimated glomerular filtration rate; FG, fasting glucose; FRS, Framingham risk score; GGT, γ-glutamyltranspeptidase; HDL-C, high-density lipoprotein cholesterol; HOMA-IR, Homeostatic model assessment of insulin resistance; hsCRP, high sensitivity C-reactive protein; HTN, hypertension; LDL-C, low-density lipoprotein cholesterol; Met Sy, metabolic syndrome; NA, not available; NAFLD, non-alcoholic fatty liver disease; NASH, nonalcoholic steatohepatitis; SBP, systolic blood pressure; SUA, serum uric acid; TC, total cholesterol; TG, triglycerides; WC, waist circumference.
