# Supplementary material for: Association of non-alcoholic fatty liver disease with major adverse cardiovascular events: A systematic review and meta-analysis
Source: Sci Rep. 2016 Sep 16;6:33386. doi: 10.1038/srep33386 (PMC5026028; doi:10.1038/srep33386)

Association of non-alcoholic fatty liver disease with major adverse cardiovascular events: A systematic review and meta-analysis

Author list:

Shunquan Wu, Fuquan Wu, Yingying Ding, Jun Hou, Jingfeng Bi, Zheng Zhang

Figure S1. Flowchart for the selection of eligible studies

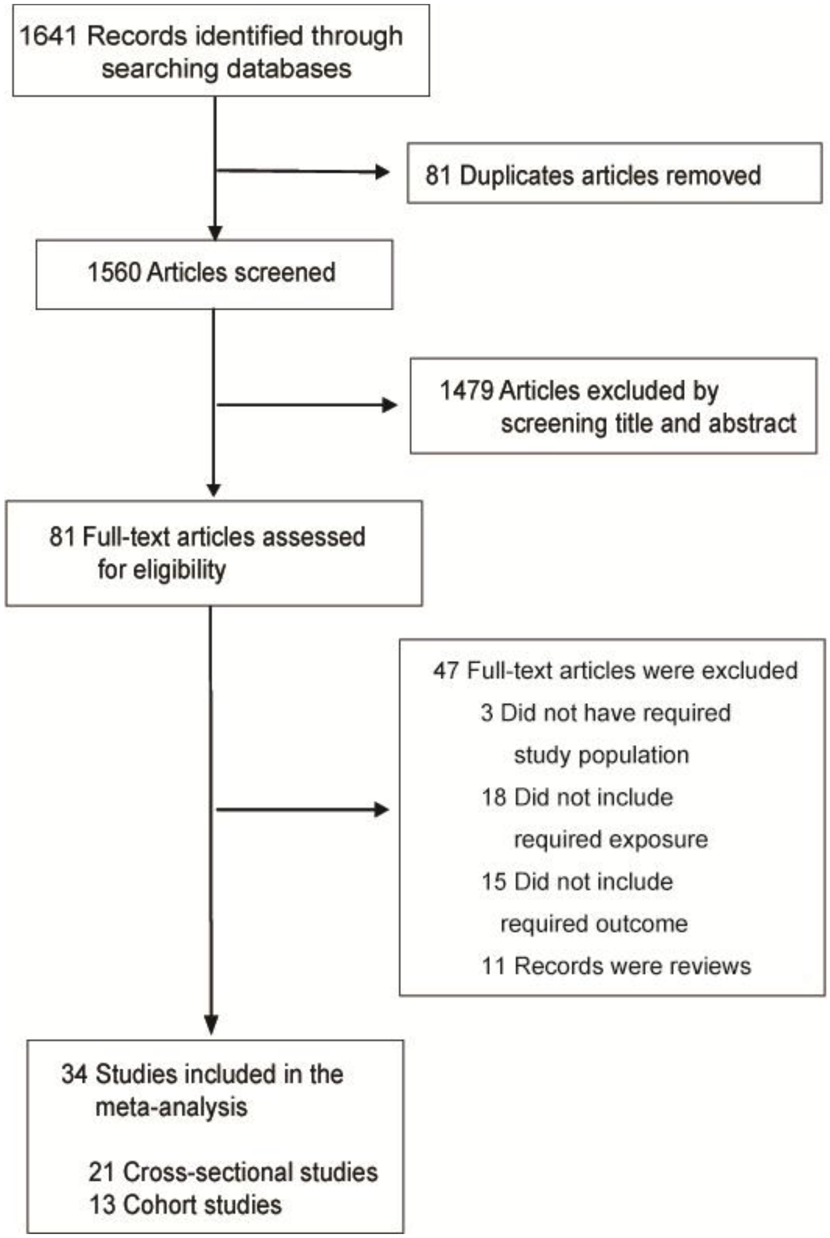

Table S2. Quality assessment of the included studies (cross-sectional studies)

Study	Selection		Comparability	Exposure	Overall quality assessment score (of a maximum of 5)
	Representativeness of the sample	Ascertainment of exposure	Comparability of groups on the basis of the design or analysis	Assessment of outcome	
Agac et al, 2013	No description	* Ultrasound	** Study controls for sex, age, BMI, waist circumference, smoking status, family history of CAD, total cholesterol, triglycerides, HDL-C, LDL-C, ALT, serum creatinine, presence of hypertension, diabetes mellitus, and MetS	* Independent blind assessment	4
Agarwal et al, 2011	No description	* Ultrasound	* Study controls for age	* Independent blind assessment	3
Arslan et al, 2007	No description	* Ultrasound	** Study controls for age, male sex, plasma LDL level, BMI, smoking history, and individual components of the metabolic syndrome	* Independent blind assessment	4
Chan et al, 2014	* Somewhat representative of the average population in the community	* Ultrasound	Study does not control for other factors	* Independent blind assessment	3
Chen et al, 2010	* Somewhat representative of the average population in the	* Ultrasound	** Study controls for sex, age, BMI, smoking, hypertension,	* Independent blind assessment	5

	community		diabetes mellitus, fasting plasma glucose, TC, TG, HDL, LDL, ALT, AST, SUA, and gallbladder stones		
Chiang et al, 2010	* Somewhat representative of the average population in the community	* Ultrasound	** Study controls for age, elevated hsCRP level, metabolic syndrome, hypertension, diabetes, and dyslipidemia	* Independent blind assessment	5
Choi et al, 2013	No description	* Ultrasound	** Study controls for age, gender, glucose, HbA1c, BMI, TC, TG, and LDL	* Independent blind assessment	4
Choi et al, 2009	* Truly representative of the average population in the community	* Ultrasound	** Study controls for age, gender, BMI, WC, and metabolic syndrome	* Independent blind assessment	5
Huang et al, 2012	* Truly representative of the average population in the community	* Ultrasound	** Study controls for age, sex, BMI, LDL-C, HOMA-IR score, regular exerciser, smoking status, drinking status, metabolic syndrome, and prior histories of cardiovascular diseases	* Independent blind assessment	5
Idilman et al, 2015	No description	*CT images	** Study controls for age, gender, LDL levels, BMI, hypertension and smoking status	* Independent blind assessment	4

Josef et al, 2013	No description	*CT images	** Study controls for gender, age, smoking habits, metabolic syndrome, diabetes, BIM, and levels of ALT, HDL and LDL-C, TG, and fasting glucose	* Independent blind assessment	4
Lopez-Suarez et al, 2011	* Truly representative of the average population in the community	* Ultrasound	** Study controls for age, sex, sedentary lifestyle, smoking status, eGFR, diabetes, BMI, HDL-C, TG, and ALT	* Independent blind assessment	5
Sun et al, 2011	No description	*CT images	** Study controls for gender, age, previous myocardial infarction, TC, and AST	* Independent blind assessment	4
Targher et al, 2006	* Truly representative of the average population in the community	* Ultrasound	** Study controls for age, sex, diabetes duration, HbA, smoking history, LDL-C, GGT levels, use of medications, and MetS	* Independent blind assessment	5
Targher et al, 2007	* Truly representative of the average population in the community	* Ultrasound	** Study controls for age, sex, BMI, smoking status, diabetes duration, A1C, LDL-C, and current use of medications	* Independent blind assessment	5
Targher et al, 2010	No description	* Ultrasound	** Study controls for age, sex, diabetes duration, HbA, smoking status, LDL-C, metabolic syndrome, BMI, SBP, HDL-C, TG, albuminuria,	* Independent blind assessment	4

			and medication use		
Targher et al, 2012	No description	* Ultrasound	** Study controls for age, gender, duration of diabetes, HbA, smoking status, alcohol consumption, physical activity level, family history of CVD, LDL-C, metabolic syndrome, BMI, SBP, HDL-C, TG, current use of anti-hypertensive, lipid-lowering or anti-platelet medications, estimated GFR, and albuminuria	* Independent blind assessment	4
Thakur et al, 2012	No description	* Ultrasound	* Study controls for generalized and abdominal obesity, metabolic syndrome, fasting insulin, dyslipidemias, systolic and diastolic blood pressure and hs-CRP	* Independent blind assessment	3
VanWagner et al, 2014	* Truly representative of the average population in the community	*CT images	** Study controls for age, race, sex, study center, income level, educational level, alcohol intake, smoking status, physical activity score, diabetes status, SBP, TC, HDL, and treatments for hypertension and dyslipidemia	* Independent blind assessment	5

Vendhan et al, 2014	* Truly representative of the average population in the community	* Ultrasound	* Study controls for age, diabetes, hypercholesterolemia, HOMA-IR, and hypertension in some but not all the analyses	* Independent blind assessment	4
Wang et al, 2015	* Truly representative of the average population in the community	* Ultrasound	** Study controls for gender, age, BMI, hyperuricemia, AST, ALT, hypercholesterolemia, hypertriglyceridemia, and fasting plasma glucose	No description	4

Table S3. Quality assessment of the included studies (cohort studies)

Study	Selection				Comparability	Outcome			Overall quality assessment score (of a maximum of 9)
	Representativeness of the exposed cohort	Selection of the non exposed cohort	Ascertainment of exposure	Demonstration that outcome of interest was not present at start of study	Comparability of cohorts on the basis of the design or analysis	Assessment of outcome	Was follow-up long enough for outcomes to occur	Adequacy of follow up of cohorts	
Adams et al, 2010	* Trulyrepresentative of the average population in the community	* Drawn from the same community as the exposed cohort	* Ultrasound	* The study demonstrated that death was not present at start of study	** Study controls for age, gender, obesity and date of diabetes diagnosis.	*Independent blind assessment	*The study selects an adequate follow up period for outcome of interest	* Subjects lost to follow up unlikely to introduce bias (100% follow up)	9
Dunn et al, 2008	* Truly representative of the average population in the community	* Drawn from the same community as the exposed cohort	* Liver biopsy	* The study demonstrated that disease was not present at start of study	* Study controls for age, gender, race, SBP, DBP, WC, TC, HDL, TG, smoking, CRP, daily alcohol, physical activity,	*Independent blind assessment	*The study selects an adequate follow up period for outcome of interest	* Subjects lost to follow up unlikely to introduce bias (100% follow up)	8

					diabetes, and HMG-CoA reductase inhibitor use in most but not all the analyses				
Ekstedt et al, 2015	* Somewhat representative of the average population in the community	* Drawn from the same community as the exposed cohort	* Liver biopsy	The study did not demonstrate that disease was not present at start of study	The study did not report the factors that controlled for	* Independent blind assessment	*The study selects an adequate follow up period for outcome of interest	* Subjects lost to follow up unlikely to introduce bias (100% follow up)	6
Hamaguchi et al, 2007	* Somewhat representative of the average population in the community	* Drawn from the same community as the exposed cohort	* Ultrasound	The study did not demonstrate that disease was not present at start of study	** Study controls for age, smoking, SBP, LDL-C, and metabolic syndrome	Self-reported	*The study selects an adequate follow up period for outcome of interest	Subjects lost to follow up likely to introduce bias (68% follow up)	6
Lazo et al, 2011	* Truly representative of the average population in the community	* Drawn from the same community as the exposed	* Ultrasound	* The study demonstrated that disease was not present at start of study	** Study controls for sex, race, education, smoking, alcohol	*Documented	*The study selects an adequate follow up period for outcome of	* Subjects lost to follow up unlikely to introduce bias (78% follow up)	9

		cohort			consumption, physical activity, BMI, hypertension, hypercholesterolaemia, and diabetes		interest		
Ong et al, 2008	* Truly representative of the average population in the community	* Drawn from the same community as the exposed cohort	* Liver enzyme	* The study demonstrated that disease was not present at start of study	** Study controls for age, gender, race, education, income, BIM, HTN, and DM	*Documented	*The study selects an adequate follow up period for outcome of interest	* Subjects lost to follow up unlikely to introduce bias (88% follow up)	9
Ryoo et al, 2014 a	* Truly representative of the average population in the community	* Drawn from the same community as the exposed cohort	* Ultrasound	* The study demonstrated that disease was not present at start of study	** Study controls for age, HDL-C, log (hsCRP), serum creatinine, recent smoking status, regular exercise, MetS and diabetes mellitus	* Independent blind assessment	The study does not select an adequate follow up period for outcome of interest	* Subjects lost to follow up unlikely to introduce bias (79% follow up)	8
Ryoo et	* Truly	* Drawn	* Ultrasound	* The study	** Study	*	The study	* Subjects	8

al, 2014 b	representative of the average population in the community	from the same community as the exposed cohort		demonstrated that disease was not present at start of study	controls for age, BMI, TG, serum creatinine, AST, ALT, GGT, recent smoking status, regular exercise and diabetes mellitus	Independent blind assessment	does not select an adequate follow up period for outcome of interest	lost to follow up unlikely to introduce bias (78% follow up)	
Stepanova et al, 2013	No description	No description	* Liver biopsy	* The study demonstrated that disease was not present at start of study	** Study controls for age, gender, race, obesity, diabetes, and hyperlipidemia	*Documented	*The study selects an adequate follow up period for outcome of interest	Subjects lost to follow up likely to introduce bias (60% follow up)	6
Stepanova et al, 2012	* Truly representative of the average population in the community	* Drawn from the same community as the exposed cohort	* Ultrasound	The study does not demonstrate that disease was not present at start of study	** Study controls for age, sex, race, obesity, diabetes mellitus, smoking, and family history of CVD	* Independent blind assessment	*The study selects an adequate follow up period for outcome of interest	Subjects lost to follow up likely to introduce bias (58% follow up)	7

Sung et al, 2009	* Truly representative of the average population in the community	* Drawn from the same community as the exposed cohort	* Ultrasound	The study does not demonstrate that disease was not present at start of study	* Study controls for age, BMI, smoking and exercise habits	* Independent blind assessment	The study does not select an adequate follow up period for outcome of interest	Subjects lost to follow up likely to introduce bias (54% follow up)	5
Sung et al, 2014	* Truly representative of the average population in the community	* Drawn from the same community as the exposed cohort	* Images	* The study demonstrated that disease was not present at start of study	** Study controls for age, sex, alcohol consumption, smoking status, exercise, SBP, BMI, diabetes status, GGT, HOMA-IR, eGFR, and change in BMI	* Independent blind assessment	*The study selects an adequate follow up period for outcome of interest	* Subjects lost to follow up unlikely to introduce bias (73% follow up)	9
Wong et al, 2011	No description	* Drawn from the same community as the exposed	* Ultrasound	* The study demonstrated that disease was not present at start of study	** Study controls for age, gender, smoking, alcohol, diabetes,	* Independent blind assessment	The study does not select an adequate follow up period for	* Subjects lost to follow up unlikely to introduce bias (100% follow up)	7

		cohort			hypertension, SBP, DBP, BMI, WC, fasting glucose, TC, HDL-C, LDL-C, TG, creatinine, and ALT		outcome of interest		

Table S4. Weighted mean difference in baseline risk factor levels among the included population, compared NAFLD participants with non-NAFLD participants

	**Number of comparisons in included studies**	**Number of population involved**	**Pooled WMD (95% CI)**	**P value**
Body mass index (kg/m^2)	27	102,080	2.82 (2.43 to 3.21)	<0.001
Waist circumference (cm)	18	71,109	8.62 (7.70 to 9.54)	<0.001
Systolic blood pressure (mmHg)	21	108,307	6.09 (4.82 to 7.35)	<0.001
Diastolic blood pressure (mmHg)	22	108,706	3.77 (2.83 to 4.71)	<0.001
Total cholesterol (mg/dl)	19	83,903	11.57 (8.54 to 14.61)	<0.001
HDL cholesterol (mg/dl)	27	101,642	-5.62 (-6.63 to -4.62)	<0.001
LDL cholesterol (mg/dl)	23	76,125	7.62 (4.13 to 11.11)	<0.001
Triglycerides (mg/dl)	28	110,278	52.27 (45.62 to 58.91)	<0.001
Fasting glucose (mg/dl)	22	106,481	8.34 (7.00 to 9.69)	<0.001
Alanine aminotransferase (IU/L)	24	87,242	14.03 (10.98 to 17.08)	<0.001
Aspartate aminotransferase (IU/L)	22	86,550	6.04 (4.48 to 7.60)	<0.001
γ -glutamyltranspeptidase (IU/L)	14	74,040	13.32 (9.88 to 16.76)	<0.001
Mean CIMT (mm)	6	9,428	0.06 (0.02 to 0.11)	0.010

CIMT, carotid intimal-medial thickness; HDL, high-density lipoprotein; LDL, low-density lipoprotein; NAFLD, non-alcoholic fatty liver disease; WMD, weighted mean difference

Table S5. Results of subgroup analyses

Outcome	Item Assessed in Analysis	Study Feature	Cross-sectional Studies<br>Pooled OR (95% CI), I^2 statistics (%), *P*-value for the heterogeneity *Q* test, number of comparisons in included studies (*n*)	Cohort Studies<br>Pooled HR (95% CI), I^2 statistics (%), *P*-value for the heterogeneity *Q* test, number of comparisons in included studies (*n*)
**Overall mortality in NAFLD vs. non-NAFLD**	**Study design**	**Population-based**	No study	1.13 (0.92-1.39); I^2=68.3%, *P*=0.013; n=5
		**Hospital-based**	No study	1.23 (1.05-1.44); I^2=0.0%, *P*=0.482; n=2
	**Mean age**	**⩾50 years**	No study	1.59 (0.93-2.72); I^2=41.6%, *P*=0.191; n=2
		**<50 years**	No study	1.17 (0.90-1.52); I^2=73.7%, *P*=0.010; n=4
	**Ethnicity**	**Non-Asian**	No study	1.14 (0.99-1.32); I^2=65.4%, *P*=0.008; n=7
		**Asian**	No study	No study
	**Presence of diabetes**	**Diabetic participants**	No study	2.20 (1.10-4.20); n=1
		**Non-diabetic participants**	No study	1.15 (0.91-1.46); n=1
		**Combined**	No study	1.10 (0.94-1.29); I^2=66.1%, *P*=0.019; n=5
	**Study quality[a]**	**High**	No study	1.13 (0.92-1.39); I^2=68.3%, *P*=0.013; n=5
		**Relatively low**	No study	1.23 (1.05-1.44); I^2=0.0%, *P*=0.482; n=2
	**Adjustment for age *and* BMI/obesity *and* smoking**	**Studies adjusting**	No study	No study

		**Studies not adjusting**	No study	1.14 (0.99-1.32); I^2=65.4%, P=0.008; n=7
**CVD mortality in NAFLD vs. non-NAFLD**	**Study design**	**Population-based**	No study	0.99 (0.75-1.30); I^2=57.2%, P=0.022; n=8
		**Hospital-based**	No study	1.45 (1.13-1.87); I^2=0.0%, P=0.541; n=2
	**Mean age**	**⩾50 years**	No study	0.92 (0.51-1.67); I^2=0.0%, P=0.903; n=3
		**<50 years**	No study	1.14 (0.86-1.53); I^2=76.2%, P<0.001; n=7
	**Ethnicity**	**Non-Asian**	No study	1.10 (0.86-1.41); I^2=64.9%, P=0.002; n=10
		**Asian**	No study	No study
	**Presence of diabetes**	**Diabetic participants**	No study	1.00 (0.48-2.07); I^2=0.0%, P=0.788; n=2
		**Non-diabetic participants**	No study	1.32 (0.89-1.96); n=1
		**Combined**	No study	1.09 (0.80-1.49); I^2=74.9%, P=0.001; n=7
	**Study quality[a]**	**High**	No study	1.32 (0.64-2.72); I^2=74.1%, P=0.004; n=5
		**Relatively low**	No study	1.08 (0.85-1.38); I^2=58.1%, P=0.049; n=5
	**Adjustment for age *and* BMI/obesity *and* smoking**	**Studies adjusting**	No study	0.92 (0.77-1.10); I^2=0.0%, P=0.667; n=3
		**Studies not adjusting**	No study	1.31 (0.88-1.96); I^2=72.0%, P=0.002; n=7
**CVD incidence in NAFLD vs. non-NAFLD**	**Study design**	**Population-based**	No study	1.21 (1.09-1.35); I^2=0.0%, P=0.996; n=4
		**Hospital-based**	1.81 (1.23-2.66); I^2=79.8%, P<0.001; n=6	4.24 (2.16-8.33); I^2=0.0%, P=0.797; n=3

	**Mean age**	**⩾50 years**	1.29 (0.99-1.68); I^2=60.7%, P=0.079; n=3	1.17 (0.69-1.98); n=1
		**<50 years**	4.19 (1.40-12.60); I^2=77.2%, *P*=0.012; n=3	1.43 (1.10-1.85); I^2=62.4%, *P*=0.021; n=6
	**Ethnicity**	**Non-Asian**	2.22 (1.23-4.01); I^2=86.6%, *P*<0.001; n=4	1.21 (1.09-1.35); I^2=0.0%, *P*=0.996; n=4
		**Asian**	1.51 (0.96-2.37); I^2=53.1%, *P*=0.144; n=2	4.24 (2.16-8.33); I^2=0.0%, *P*=0.797; n=3
	**Presence of diabetes**	**Diabetic participants**	1.83 (1.16-2.90); I^2=82.5%, *P*<0.001; n=5	No study
		**Non-diabetic participants**	No study	4.24 (2.16-8.33); I^2=0.0%, *P*=0.797; n=3
		**Combined**	1.89 (1.23-2.91); n=1	1.21 (1.09-1.35); I^2=0.0%, *P*=0.996; n=4
	**Study quality[a]**	**High**	1.45 (1.05-1.99); I^2=73.2%, *P*=0.024; n=3	1.17 (0.69-1.98); n=1
		**Relatively low**	3.70 (0.86-15.93); I^2=87.0%, *P*<0.001; n=3	1.43 (1.10-1.85); I^2=62.4%, *P*=0.021; n=6
	**Adjustment for age *and* BMI/obesity *and* smoking**	**Studies adjusting**	3.99 (1.13-14.07); I^2=84.6%, *P*=0.002; n=3	1.22 (1.09-1.36); I^2=0.0%, *P*=0.977; n=3
		**Studies not adjusting**	1.31 (0.95-1.81); I^2=58.5%, *P*=0.090; n=3	2.80 (1.18-6.68); I^2=67.2%, *P*=0.028; n=4
**CAD incidence in NAFLD vs. non-NAFLD**	**Study design**	**Population-based**	1.94 (0.72-5.23); I^2=59.1%, *P*=0.118; n=2	No study
		**Hospital-based**	1.87 (1.45-2.39); I^2=82.2%, *P*<0.001; n=13	2.31 (1.46-3.65); n=1
	**Mean age**	**⩾50 years**	2.76 (1.51-5.04); I^2=79.4%, *P*<0.001; n=8	2.31 (1.46-3.65); n=1
		**<50 years**	1.33 (1.19-1.49); I^2=26.1%, *P*=0.230; n=7	No study
	**Ethnicity**	**Non-Asian**	No study	No study
		**Asian**	1.87 (1.47-2.37); I^2=80.2%, *P*<0.001; n=15	2.31 (1.46-3.65); n=1
	**Presence of diabetes**	**Diabetic participants**	1.55 (0.92-2.62); I^2=31.4%, *P*=0.233; n=3	No study

		**Non-diabetic participants**	2.37 (1.34-4.17); I^2=30.3%, P=0.230; n=4	No study
		**Combined**	1.85 (1.38-2.48); I^2=88.0%, P<0.001; n=8	2.31 (1.46-3.65); n=1
	**Study quality[a]**	**High**	1.30 (1.19-1.41); I^2=0.0%, P=0.524; n=5	No study
		**Relatively low**	2.56 (1.55-4.23); I^2=76.0%, P<0.001; n=10	2.31 (1.46-3.65); n=1
	**Adjustment for age *and* BMI/obesity *and* smoking**	**Studies adjusting**	2.78 (1.64-4.70); I^2=39.8%, P=0.141; n=6	2.31 (1.46-3.65); n=1
		**Studies not adjusting**	1.66 (1.29-2.14); I^2=84.8%, P<0.001; n=9	No study
**Hypertension incidence in NAFLD vs. non-NAFLD**	**Study design**	**Population-based**	1.71 (1.10-2.65); n=1	No study
		**Hospital-based**	1.23 (1.12-1.35); I^2=0.0%, P=0.934; n=3	1.16 (1.06-1.27); I^2=55.9%, P=0.059; n=5
	**Mean age**	**⩾50 years**	1.71 (1.10-2.65); n=1	No study
		**<50 years**	1.23 (1.12-1.35); I^2=0.0%, P=0.934; n=3	1.16 (1.06-1.27); I^2=55.9%, P=0.059; n=5
	**Ethnicity**	**Non-Asian**	1.71 (1.10-2.65); n=1	No study
		**Asian**	1.23 (1.12-1.35); I^2=0.0%, P=0.934; n=3	1.16 (1.06-1.27); I^2=55.9%, P=0.059; n=5
	**Presence of diabetes**	**Diabetic participants**	No study	No study
		**Non-diabetic participants**	1.23 (1.12-1.35); I^2=0.0%, P=0.934; n=3	No study
		**Combined**	1.71 (1.10-2.65); n=1	1.16 (1.06-1.27); I^2=55.9%, P=0.059; n=5
	**Study quality[a]**	**High**	1.71 (1.10-2.65); n=1	1.16 (1.06-1.27); I^2=55.9%, P=0.059; n=5
		**Relatively low**	1.23 (1.20-1.35); I^2=0.0%, P=0.934; n=3	No study

	**Adjustment for age *and* BMI/obesity *and* smoking**	**Studies adjusting**	1.71 (1.10-2.65); n=1	1.09 (1.03-1.16); I^2=0.0%, *P*=0.515; n=3
		**Studies not adjusting**	1.23 (1.20-1.35); I^2=0.0%, *P*=0.934; n=3	1.34 (0.99-1.82); I^2=75.2%, *P*=0.044; n=2
**Atherosclerosis incidence in NAFLD vs. non-NAFLD**	**Study design**	**Population-based**	1.28 (1.14-1.43); I^2=0.0%, *P*=0.480; n=4	No study
		**Hospital-based**	4.80 (1.80-12.80); n=1	No study
	**Mean age**	**⩾50 years**	1.28 (1.14-1.43); I^2=0.0%, *P*=0.480; n=4	No study
		**<50 years**	4.80 (1.80-12.80); n=1	No study
	**Ethnicity**	**Non-Asian**	1.15 (0.86-1.54); I^2=34.0%, *P*=0.218; n=2	No study
		**Asian**	1.48 (1.08-2.03); I^2=69.9%, *P*=0.036; n=3	No study
	**Presence of diabetes**	**Diabetic participants**	No study	No study
		**Non-diabetic participants**	4.80 (1.80-12.80); n=1	No study
		**Combined**	1.28 (1.14-1.43); I^2=0.0%, *P*=0.480; n=4	No study
	**Study quality[a]**	**High**	1.28 (1.14-1.43); I^2=0.0%, *P*=0.480; n=4	No study
		**Relatively low**	4.80 (1.80-12.80); n=1	No study
	**Adjustment for age *and* BMI/obesity *and* smoking**	**Studies adjusting**	1.31 (1.15-1.50); I^2=0.0%, *P*=0.796; n=2	No study
		**Studies not adjusting**	1.33 (0.96-1.84); I^2=78.2%, *P*=0.010; n=3	No study

**Overall mortality in NASH vs. non-NASH**	**Study design**	**Population-based**	No study	0.80 (0.52-1.22); n=1
		**Hospital-based**	No study	1.56 (0.94-2.58); I^2=89.5%, P<0.001; n=4
	**Mean age**	**⩾50 years**	No study	1.13 (0.74-1.72); n=1
		**<50 years**	No study	1.44 (0.80-2.57); I^2=89.3%, P<0.001; n=4
	**Ethnicity**	**Non-Asian**	No study	1.37 (0.86-2.19); I^2=86.4%, P<0.001; n=5
		**Asian**	No study	No study
	**Presence of diabetes**	**Diabetic participants**	No study	No study
		**Non-diabetic participants**	No study	No study
		**Combined**	No study	1.37 (0.86-2.19); I^2=86.4%, P<0.001; n=5
	**Study quality**[a]	**High**	No study	0.80 (0.52-1.23); n=1
		**Relatively low**	No study	1.56 (0.94-2.59); I^2=85.9%, P<0.001; n=4
	**Adjustment for age *and* BMI/obesity *and* smoking**	**Studies adjusting**	No study	No study
		**Studies not adjusting**	No study	1.37 (0.86-2.19); I^2=86.4%, P<0.001; n=5
**CVD mortality in NASH vs. non-NASH**	**Study design**	**Population-based**	No study	0.59 (0.29-1.20); n=1
		**Hospital-based**	No study	1.41 (0.61-3.22); I^2=83.8%, P<0.001; n=4
	**Mean age**	**⩾50 years**	No study	0.51 (0.23-1.12); n=1

	**<50 years**	No study	1.44 (0.65-3.20); I^2=83.2%, P<0.001; n=4
**Ethnicity**	**Non-Asian**	No study	1.18 (0.57-2.48); I^2=83.3%, P<0.001; n=5
	**Asian**	No study	No study
**Presence of diabetes**	**Diabetic participants**	No study	No study
	**Non-diabetic participants**	No study	No study
	**Combined**	No study	1.18 (0.57-2.48); I^2=83.3%, P<0.001; n=5
**Study quality[a]**	**High**	No study	0.59 (0.29-1.20); n=1
	**Relatively low**	No study	1.41 (0.61-3.22); I^2=83.8%, P<0.001; n=4
**Adjustment for age *and* BMI/obesity *and* smoking**	**Studies adjusting**	No study	No study
	**Studies not adjusting**	No study	1.18 (0.57-2.48); I^2=83.3%, P<0.001; n=5

[a] We considered a cross-sectional study awarded 5 stars and a cohort study awarded 8 or more stars as a high quality study in current study, as no standard criteria has been established

Figure S2. Sensitivity analyses. Pooled relative risks for cardiovascular events associated with non--alcoholic fatty liver disease by omitting one study in turn.

A. Overall mortality

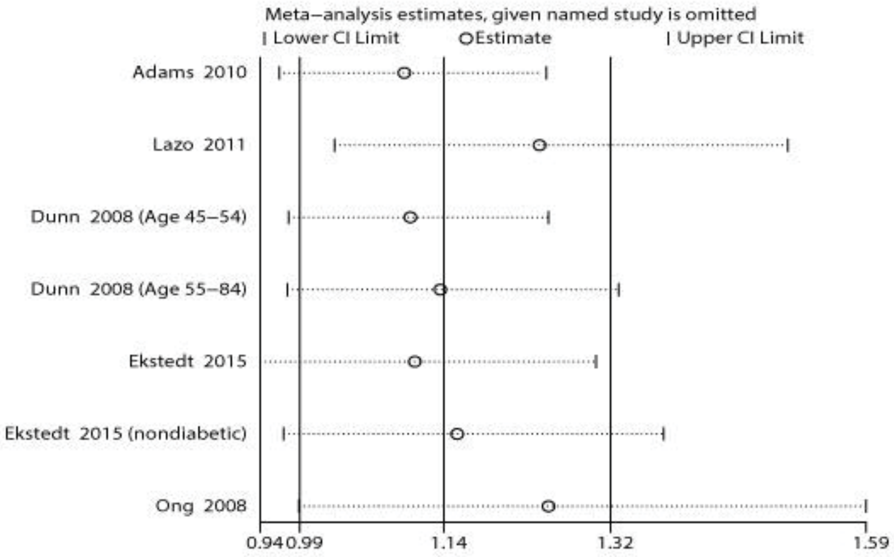

B. CVD mortality

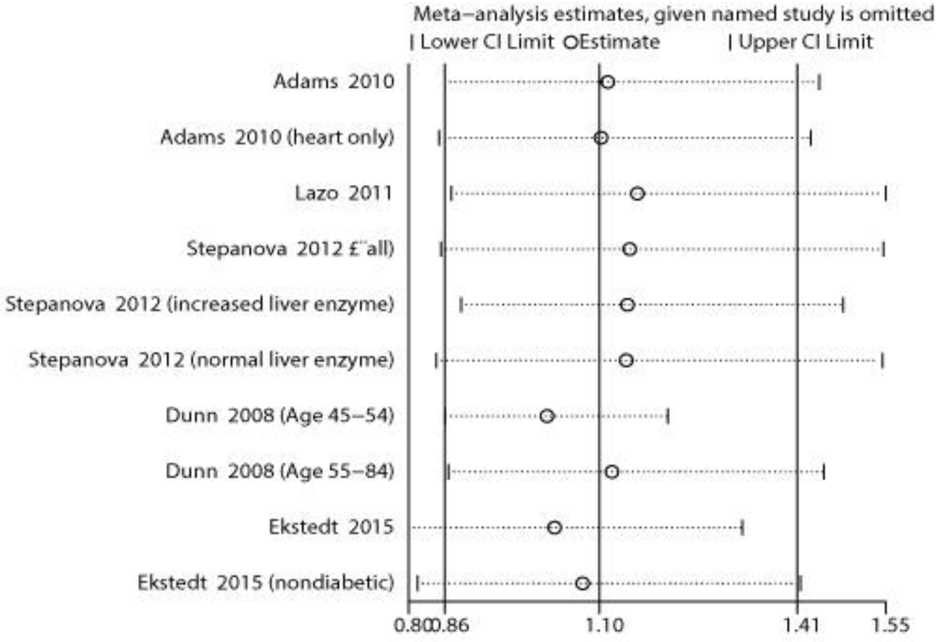

C. CVD prevalence (Cross-sectional studies)

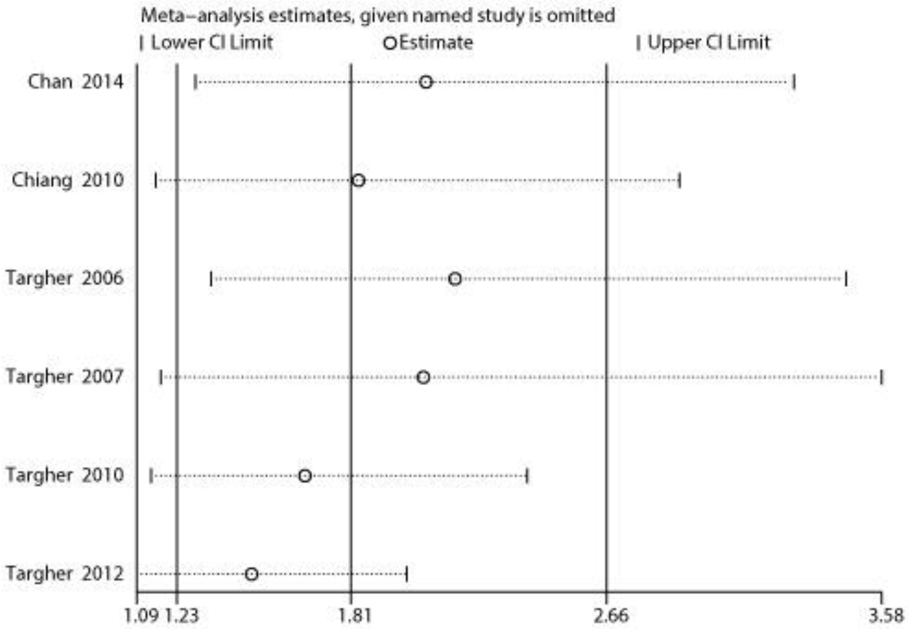

D. CVD incidence (Cohort studies)

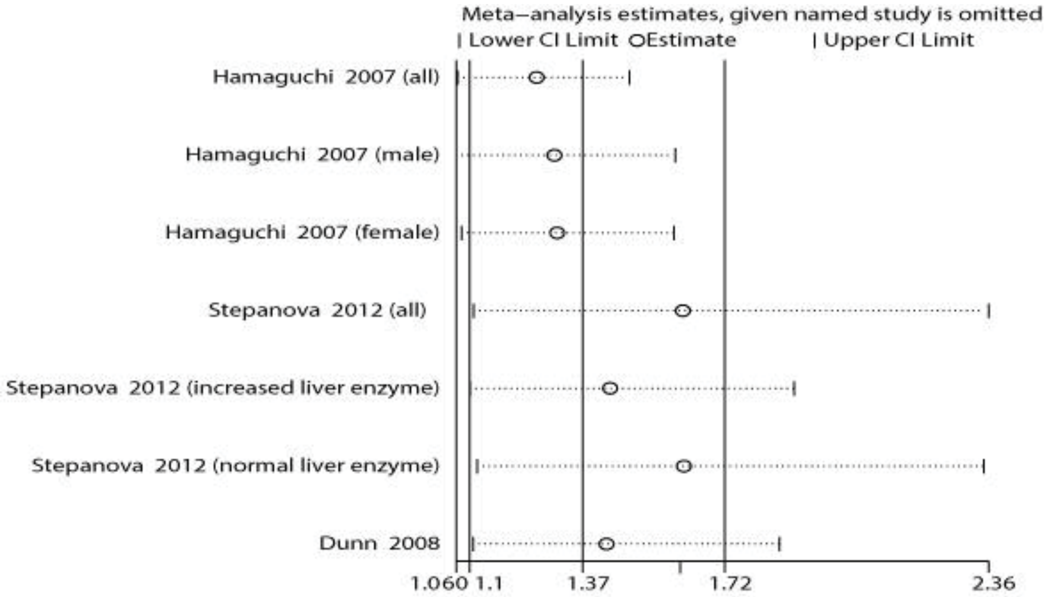

E. CAD prevalence (Cross-sectional studies)

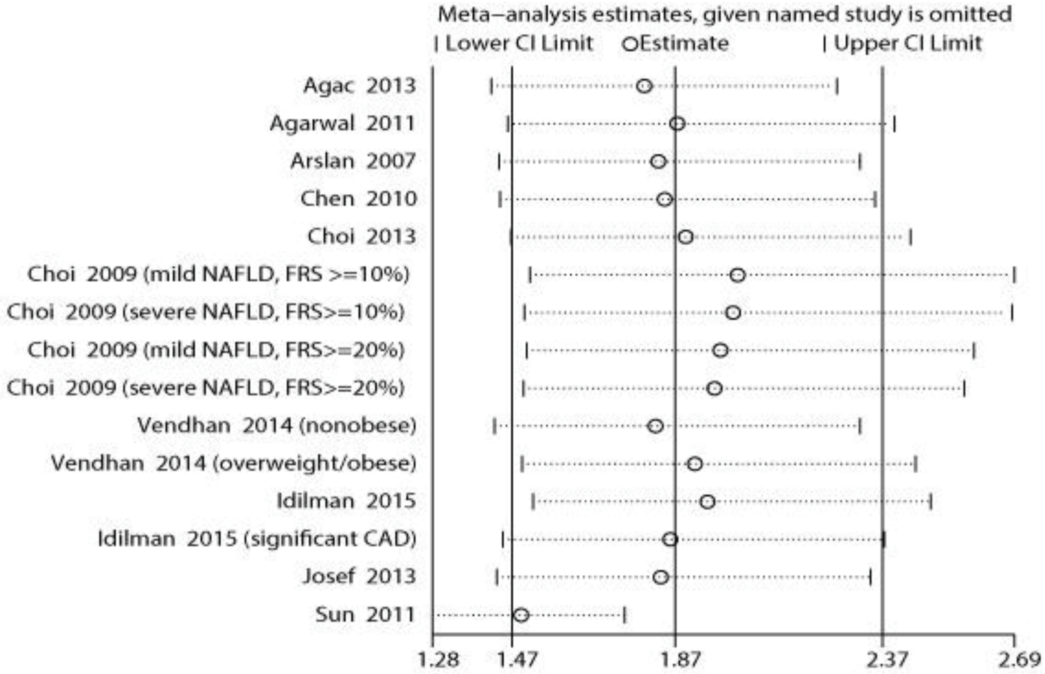

F. Hypertension prevalence (Cross-sectional studies)

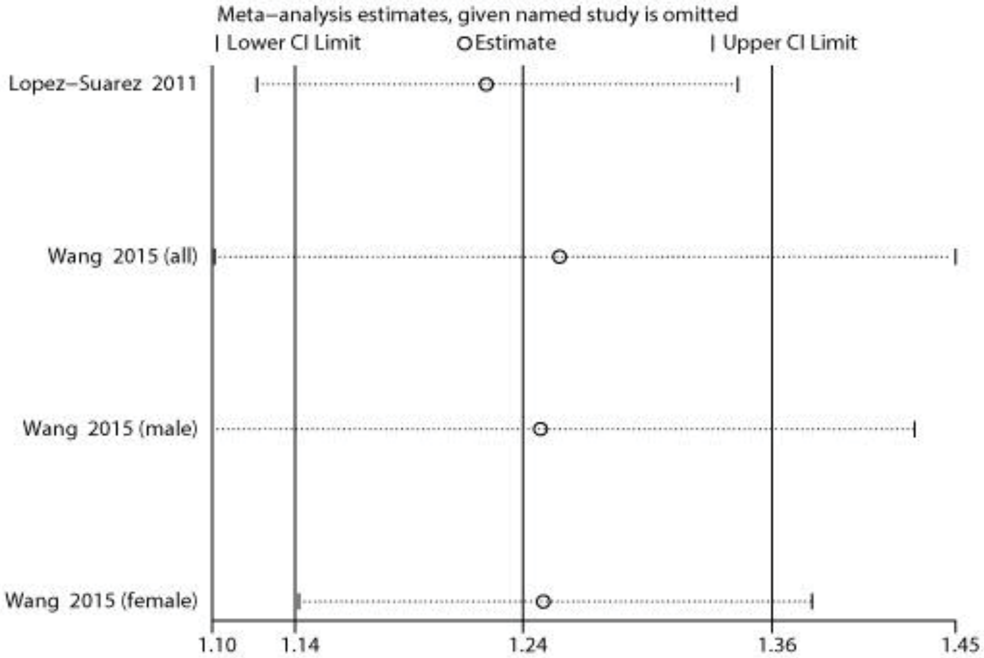

G. Hypertension incidence (Cohort studies)

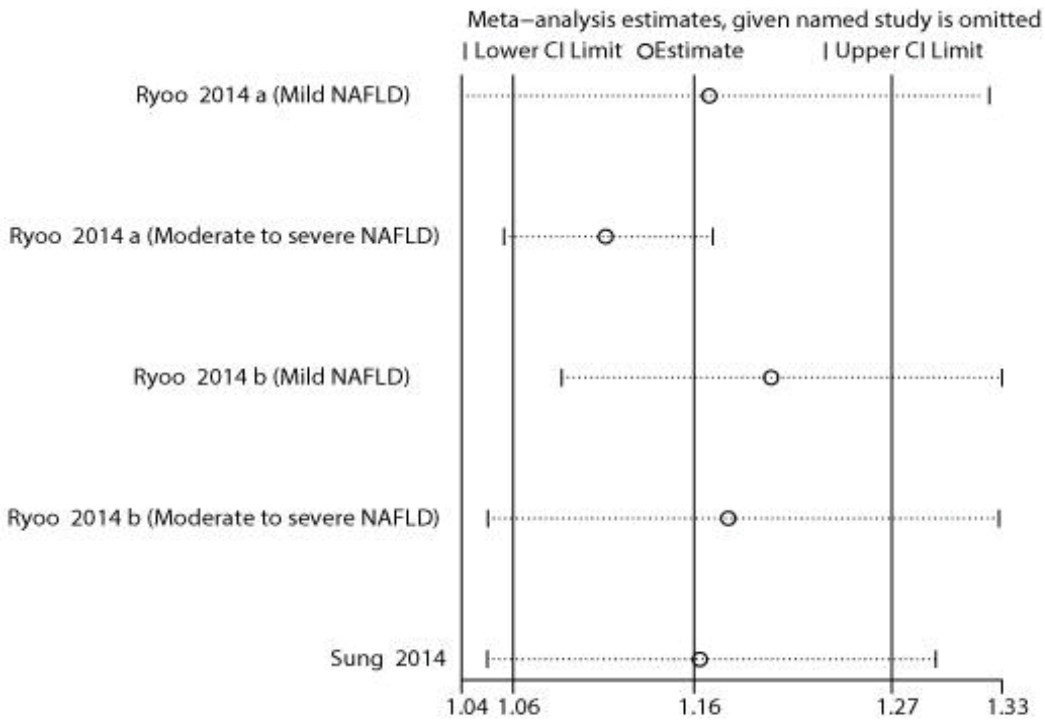

H. Atherosclerosis prevalence

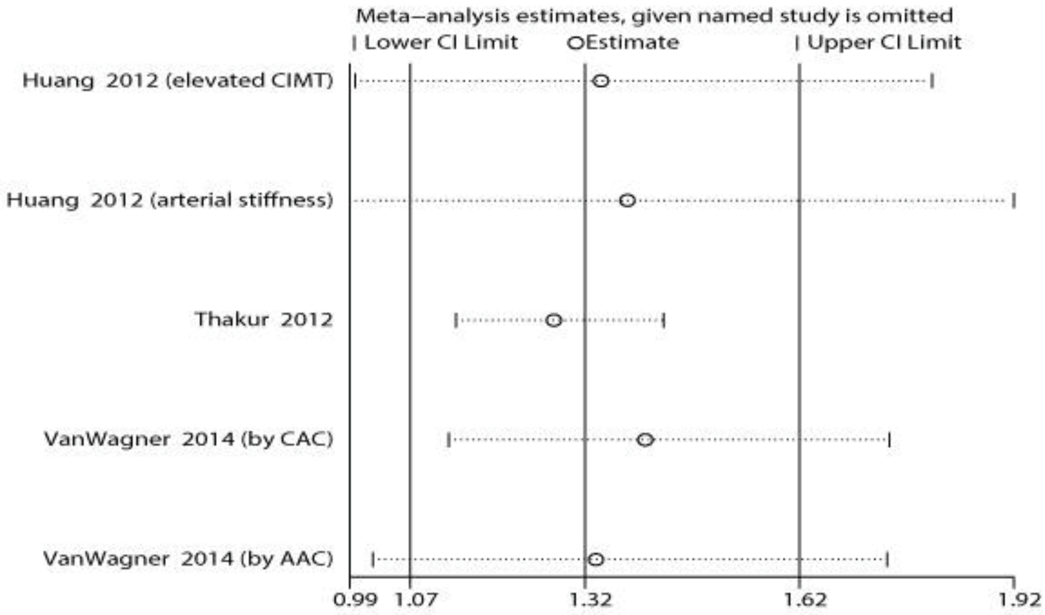

I. NASH: Overall mortality

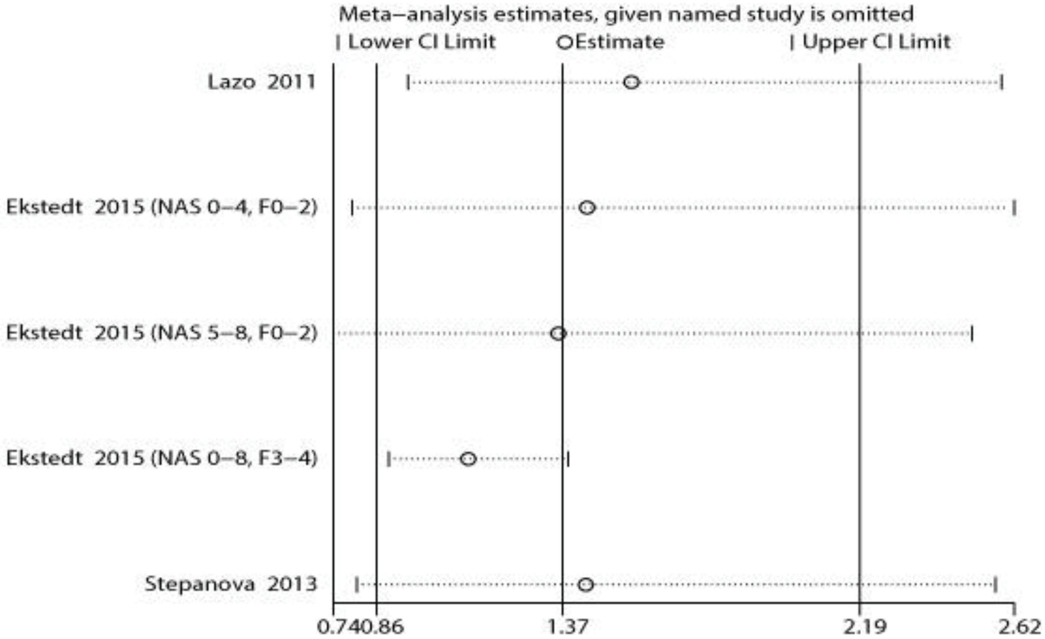

J. NASH: CVD mortality

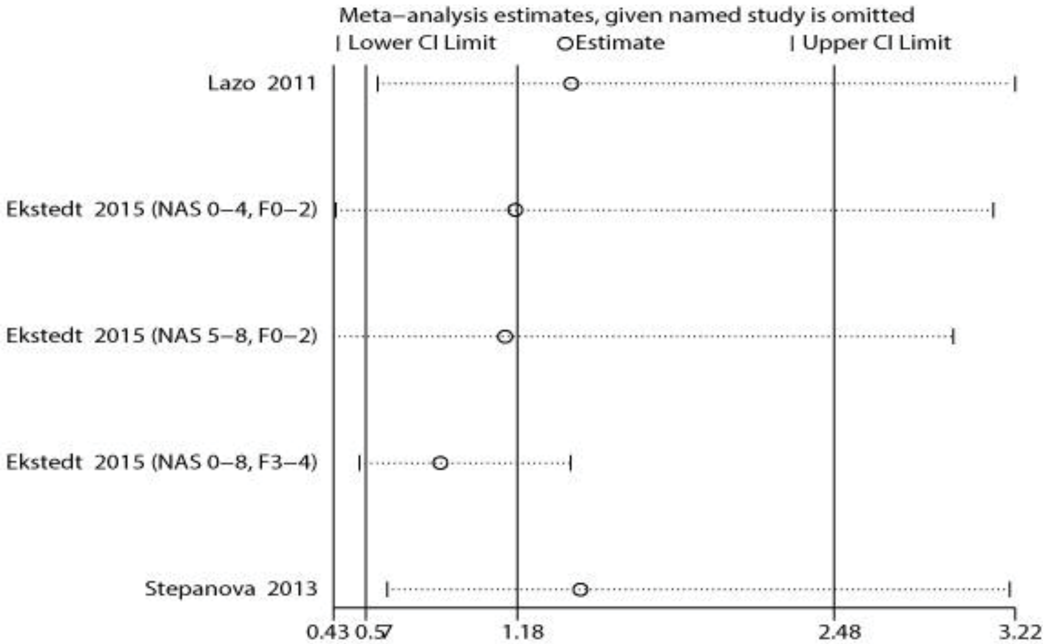

Figure S3. Funnel plots to assess publication bias

Plots show study size as a function of effect size for studies included in the meta-analysis.

A. Overall mortality

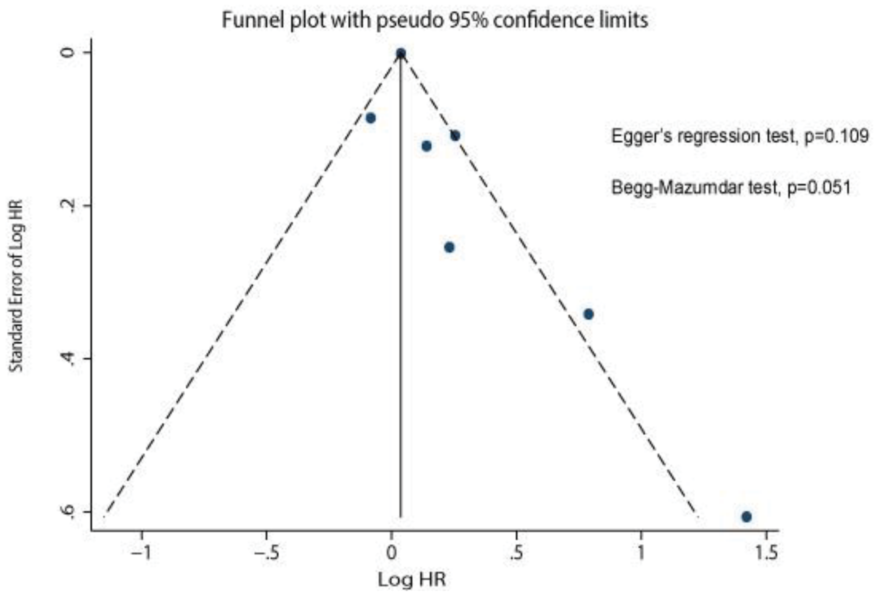

B. CVD mortality

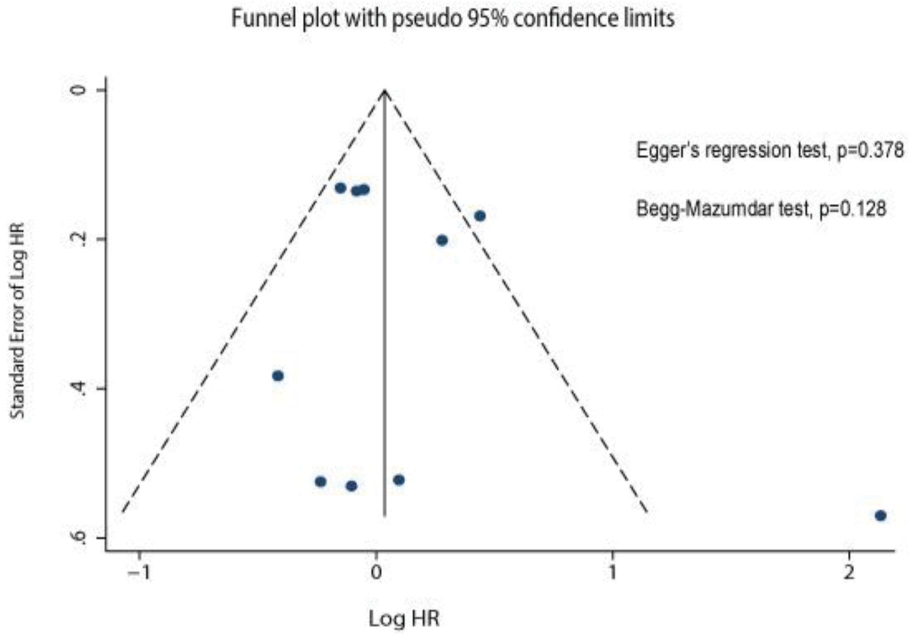

C. CVD prevalence (Cross-sectional studies)

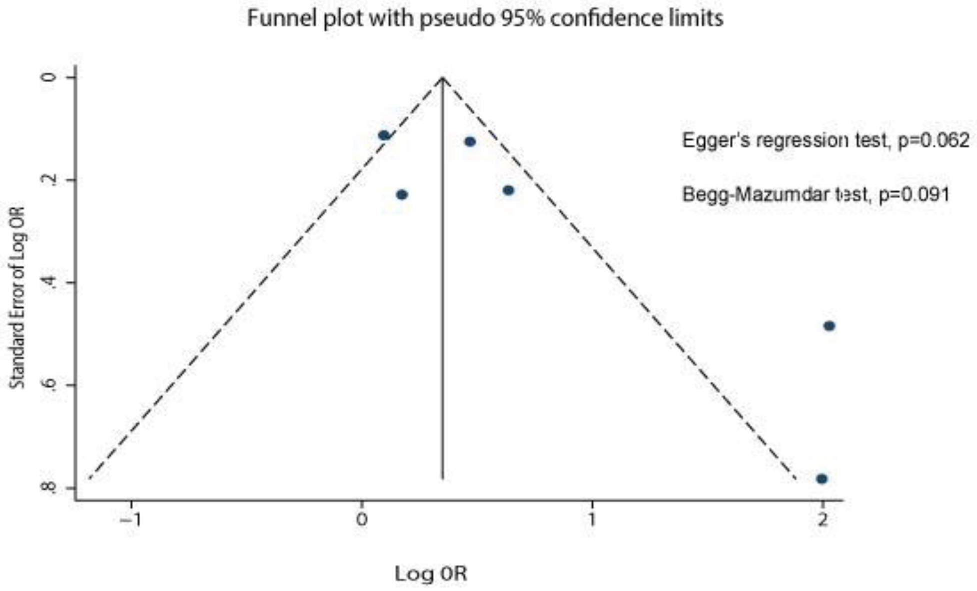

D. CVD incidence (Cohort studies)

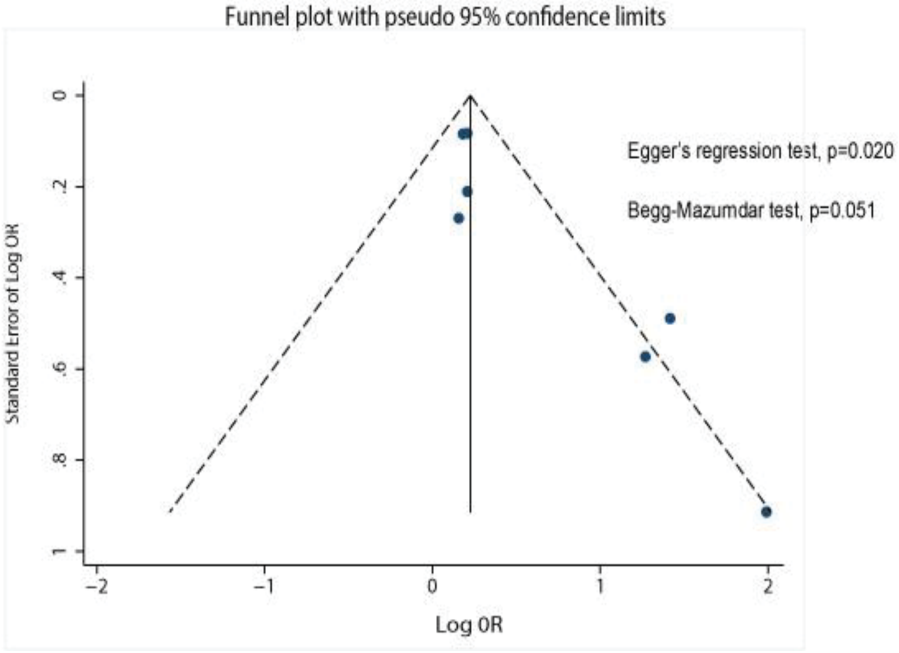

E. CVD incidence (Cohort studies) after using the trim and fill approach.
1 study was filled. Pooled HR (95% CI) after filling was 1.36 (1.06-1.74)

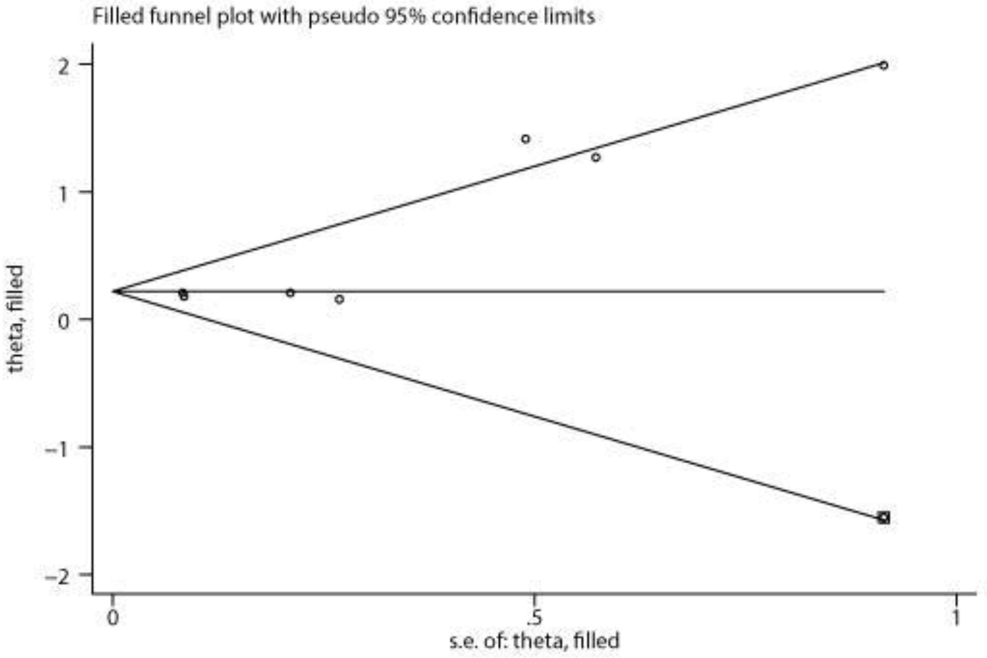

F. CAD prevalence (Cross-sectional studies)

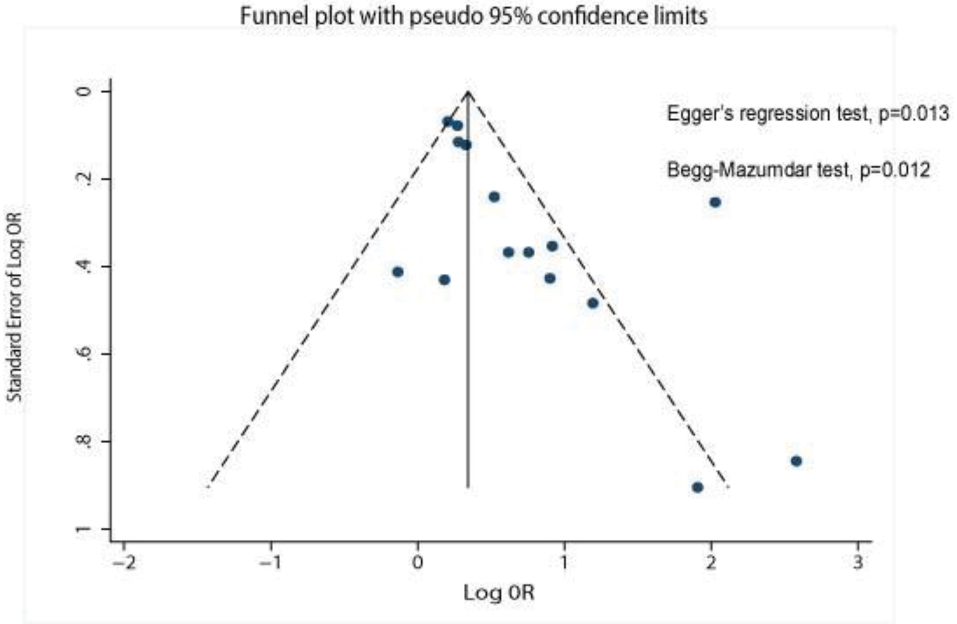

G. CAD prevalence (Cross-sectional studies) after using the trim and fill approach. 6 studies was filled. Pooled OR (95% CI) after filling was 1.36 (1.04-1.77)

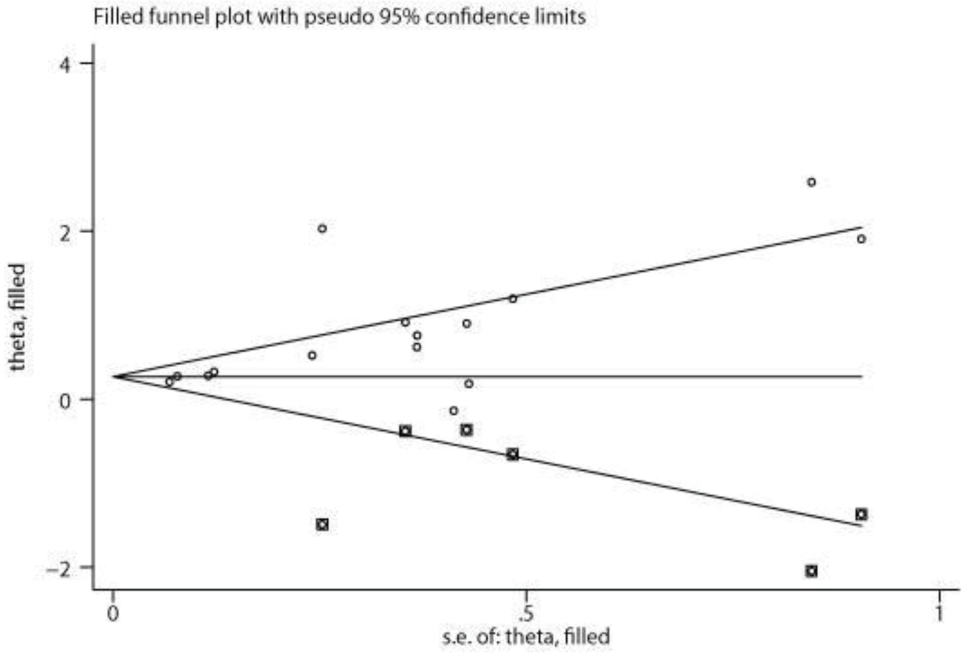

H. Hypertension prevalence (Cross-sectional studies)

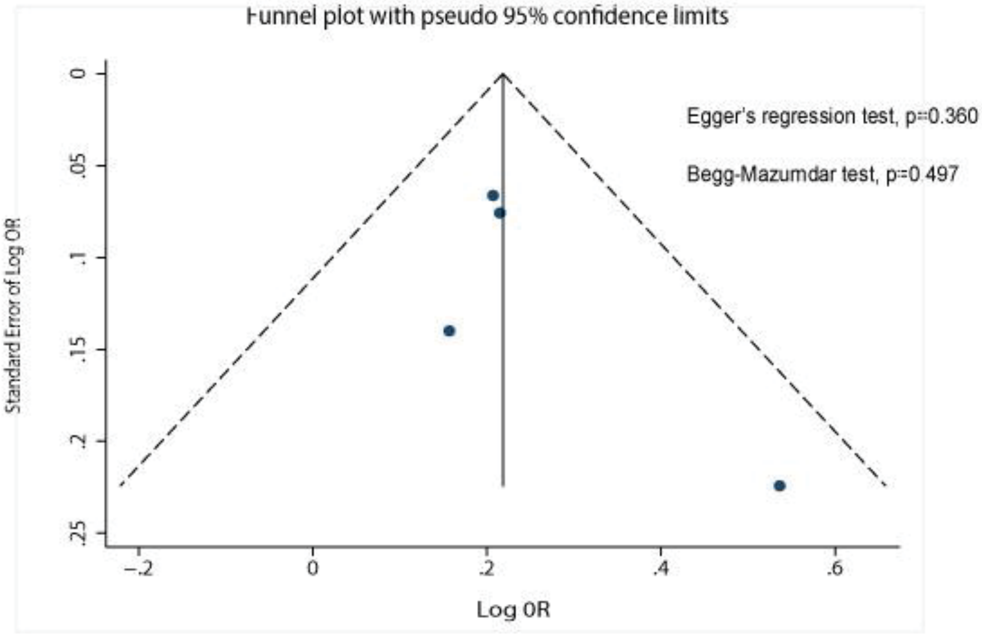

I. Hypertension incidence (Cohort studies)

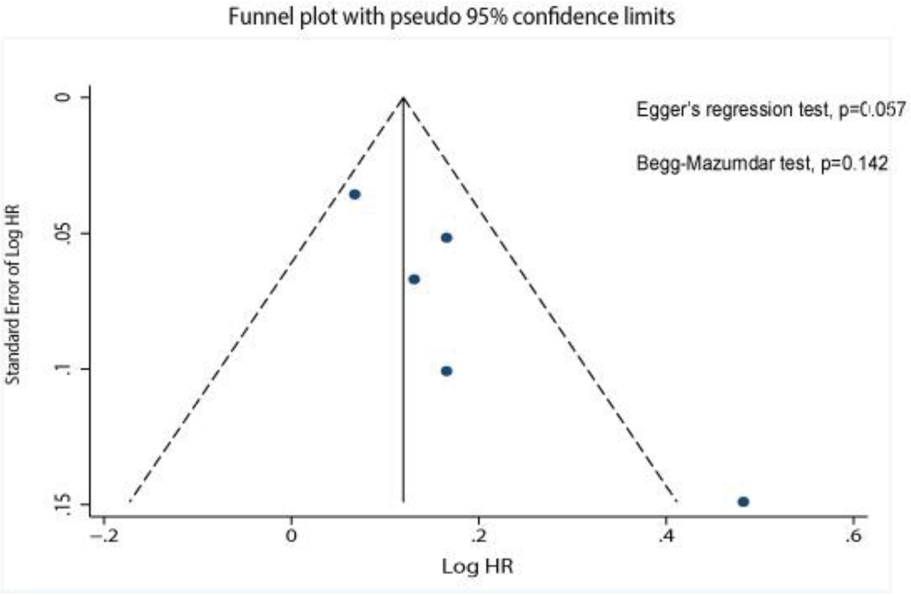

J. Atherosclerosis prevalence

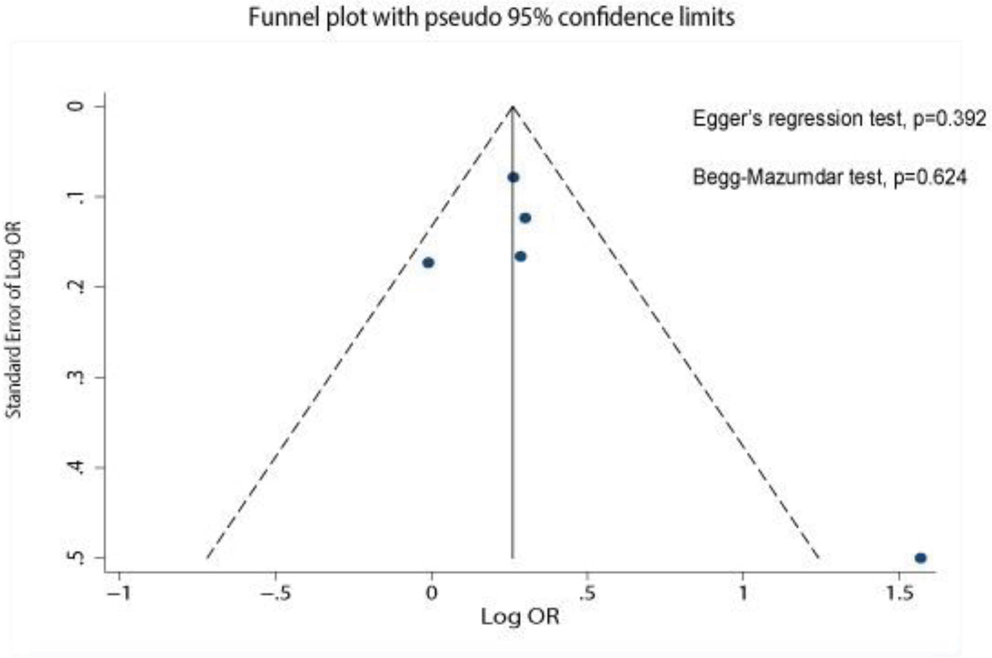

K. NASH: overall mortality

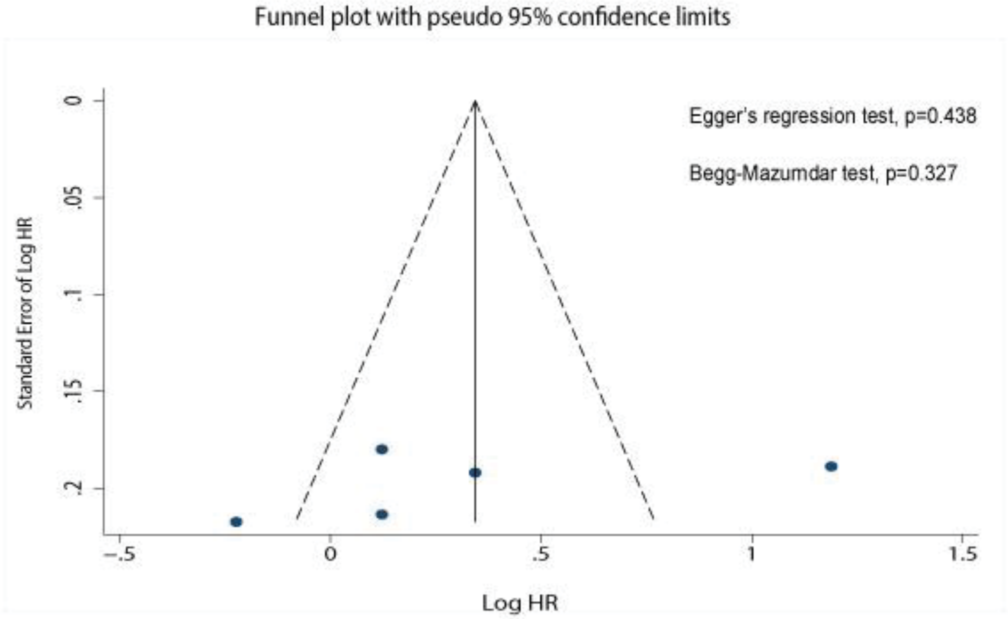

L. NASH: CVD mortality

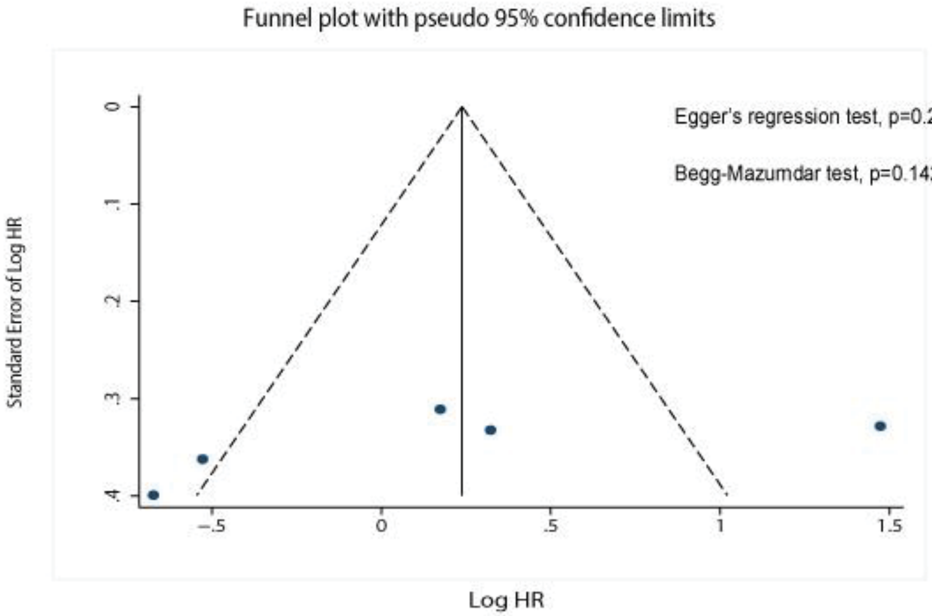

Supplement: Supplementary Information [file srep33386-s1.pdf]
